# Polarity protein AF6 functions as a modulator of necroptosis by regulating ubiquitination of RIPK1 in liver diseases

**DOI:** 10.1038/s41419-023-06170-8

**Published:** 2023-10-12

**Authors:** Wang Xinyu, Wen Qian, Wu Yanjun, Kong Jingwen, Xu Keying, Jiao Jiazheng, Zhang Haibing, Wang Kai, Xu Xiao, Zhan Lixing

**Affiliations:** 1 CAS Key Laboratory of Nutrition, Metabolism and Food Safety, Shanghai Institute of Nutrition and Health, Shanghai Institutes for Biological Sciences, University of Chinese Academy of Sciences, Chinese Academy of Sciences, Shanghai, China; 2 Key Laboratory of Integrated Oncology and Intelligent Medicine of Zhejiang Province, Department of Hepatobiliary and Pancreatic Surgery, Affiliated Hangzhou First People’s Hospital, Zhejiang University School of Medicine, Hangzhou, 310006 China

**Keywords:** Tight junctions, Necroptosis

## Abstract

AF6, a known polarity protein, contributes to the maintenance of homeostasis while ensuring tissue architecture, repair, and integrity. Mice that lack AF6 display embryonic lethality owing to cell–cell junction disruption. However, we show AF6 promotes necroptosis via regulating the ubiquitination of RIPK1 by directly interact with the intermediate domain of RIPK1, which was mediated by the deubiquitylase enzyme USP21. Consistently, while injection of mice with an adenovirus providing AF6 overexpression resulted in accelerated TNFα-induced necroptosis-mediated mortality in vivo, we observed that mice with hepatocyte-specific deletion of AF6 prevented hepatocytes from necroptosis and the subsequent inflammatory response in various liver diseases model, including non-alcoholic steatohepatitis (NASH) and the systemic inflammatory response syndrome (SIRS).Together, these data suggest that AF6 represents a novel regulator of RIPK1-RIPK3 dependent necroptotic pathway. Thus, the AF6-RIPK1-USP21 axis are potential therapeutic targets for treatment of various liver injuries and metabolic diseases.

## Introduction

Liver is a vital hub for synthesis, metabolism, and energy production and conversion in the body. In both normal physiology and human disease, cell death and inflammation are classic processes central to biological function [1–3]. Since its discovery in 2005, necroptosis has been the subject of extensively research [4]. In contrast to apoptosis, necroptosis causes rupture of the cell membrane; the resulting discharge of the cellular contents triggers substantial inflammation and immune cells activation, creating a microenvironment that is favorable for disease progression.

Polarity proteins constitute a protein family that includes both transmembrane proteins and scaffold proteins. AF6, a scaffold protein that is expressed in almost all epithelial tissues [5], is instrumental in maintaining the barrier function and the homeostasis of cell tissue structures [6]. Furthermore, *Af6* knockout mice display embryonic lethality owing to developmental defects [7, 8]. Recently, we demonstrated that AF6 contributes to blood glucose homeostasis and insulin sensitivity in liver [9], a previously unreported function of AF6. As part of that work, we showed that AF6 accumulates to abnormally high levels in two diabetic mouse models, specifically the high-fat diet (HFD) and *db/db* mouse models [9]. Notably, using treatment with oleic acid (OA) and palmitic acid (PA), we demonstrated that the expression of AF6 is directly up-regulated in primary hepatocytes exhibiting lipid accumulation [9]. Therefore, we postulated that the abnormal accumulation of AF6 in liver may be a critical step in the progression of liver metabolic diseases. Other recent work in *Drosophila* implies that Grindelwald, the first example of a tumor necrosis factor (TNF) receptor, integrates the pro apoptotic-signals from Egr and apical polarity [10] and apoptosis regulated by the tight junction-associated polarity protein Par3 is critical for mammary cell survival [11]. Crosstalk between polar protein signaling and cell death is an important mediator of most aspects of disease progression [12–14]. The involvement of polarity proteins in the molecular mechanisms underlying the regulation of cell death in necroptosis and liver metabolic disease, remains poorly understood.

In the present study (and in previous studies), we demonstrated that AF6 is upregulated during concomitant with hepatic fat accumulation both in vitro and in vivo. We further showed that the over-expression of AF6 results in the activation of TNF-induced necroptosis in mouse embryonic fibroblast (MEF) cells, primary hepatocytes, bone marrow-derived macrophages (BMDMs) and a normal fibroblast cell line (L929). In addition, we demonstrated that hepatic knockout of *Af6* prevented hepatocyte necroptosis and the potential consequences of hepatocyte necroptosis by disrupting the ubiquitination of RIPK1 through deubiquitylase enzyme ubiquitin-specific peptidase 21 (USP21). Together, these results indicated that AF6 promotes inflammation and cell death in most liver diseases, including acute liver injury, non-alcoholic steatohepatitis (NASH) and the systemic inflammatory response syndrome (SIRS). Collectively, our findings identify AF6 as a novel regulator of hepatocyte necroptosis, emphasizing the critical role of AF6 in sensitizing fatty liver deposits to TNFα-induced necroptosis, suggesting that AF6 may serve as therapeutic target for the prevention of necroptosis-related hepatic disease.

## Methods and materials

### Animals

Wild-type male C57BL/6J and *db/db* (in a C57BL/6J background) mice were purchased from Shanghai Laboratory Animal Co. (Shanghai, China). The generation of liver-specific *Af6* knockout (*Af6* ^*LKO*^) mice was described previously [9]. In brief summary, *Af6* ^*LKO*^ mice we used in this study was a liver-conditional *Af6*-KO mouse with loxP sites flanking exon 2 of *Af6*. *Af6* ^*flox/flox*^ mice were crossed with albumin-Cre mice to generate liver-specific *Af6* knockout (*Af6*^*LKO*^) offspring. All mice were males, 8–12 weeks of age, weighing 20–30 g, and randomized into experiment. Animals were maintained under a 12-h/12-h light/dark cycle at 25 °C and were provided with free access to water and food. For NASH model, mice were maintained for 16 weeks on a high-fat, high-cholesterol (HFHC) diet (TrophicDiet Cat. TP26304) consisting of 14% protein, 42% fat, 44% carbohydrates, and 0.2% cholesterol before euthanasia. For the acetaminophen (APAP)-overdose induced acute liver injury model, animals were food fasted for 14–16 h and then injected intraperitoneally with APAP (400 mg/kg) and returned to their cages (with food) for 6–12 h before euthanasia. For the TNFα-induced SIRS model, animals were injected intravenously (via a lateral tail vein) with mouse TNFα (360 μg/kg), and the core temperature was measured every hour thereafter; undead animals were euthanized at 24 h post-dose. At the respective termination time point, mice of each model were anesthetized with avertin if blood collection was required or euthanized by cervical dislocation without blood collection; the liver tissues then were collected and snap frozen in liquid nitrogen pending further analysis. All experimental procedures were approved (SINH-2020-ZLX-2) by the Institutional Animal Care and Use Committee of the Shanghai Institute of Nutrition and Health, Chinese Academy of Sciences.

### Hepatocyte isolation and culture

Primary hepatocytes were isolated by the collagenase perfusion method [15]. After anesthesia, a venous indwelling needle was inserted and fixed at the superior vena cava. The inferior vena cava then was cut, and Krebs Ringer buffer with glucose was injected to remove blood from the liver (2 ml/min). The liver then was perfused with a digestive solution containing 800U collagenase I (Worthington) in Krebs Ringer buffer with glucose for 20 min (2.5 ml/min). Following digestion, the entire liver was excised, placed in pre-chilled Dulbecco’s Modified Eagle Medium (DMEM), and stored on ice for later separation/dissociation (within 2 h of collection). The resulting suspension of liver cells was subjected to centrifugation (50 *g*, 4 °C, 10 min) on 45% Percoll (Sigma-Aldrich) to separate the live and dead cells. The live cells were recovered, resuspended in DMEM supplemented with 10% fetal bovine serum (Hyclone) and 2% penicillin/streptomycin spread on collagen-coated (by overnight incubation) cell culture plates, and grown at 37 °C with 5% CO_2_.

### Western blotting and immunoprecipitation

For western blotting and immunoprecipitation analysis, cells were lysed with NP-40 buffer (Tris-HCl 20 mM (pH 7.5), NaCl 120 mM, ethylenediaminetetraacetic acid (EDTA) 1 mM, ethylene glycol-bis-N, N, N′, N′-tetraacetic acid 1 mM, nonyl phenoxypolyethoxylethanol (NP-40) 0.5%, glycerol 10%, vanadate 2.5 mM, pepstatin A 1 mM, phenylmethylsulfonyl fluoride 1 mM, leupeptin 1 μg/mL, and aprotinin 1 μg/mL) on ice for 20 min. After centrifugation at 12,000 × *g* for 10 min at 4 °C, the protein concentrations of the supernatant were measured using an enhanced chemiluminescence kit (Pierce). The supernatants were mixed with loading buffer and denatured at 100 °C for 10 min. For immunoprecipitation, aliquots of the cell lysate containing 0.5 mg total protein were incubated with 1 μg specific antibody or immunoglobulin G and G-agarose beads. After overnight incubation at 4 °C with rotation, the mixture was washed five times with NP-40 buffer and then denatured in loading buffer at 100 °C for 10 min. To immunoprecipitate Complex I, cells were lysed in NP-40 buffer following stimulation with 100 ng/mL TNFα-FLAG (Enzo Life Science, ALX-522-009-C050). The cell lysates were then incubated with FLAG-tagged beads (Bimake, B23101) for 2 h at room temperature. The beads were collected and washed five times with NP-40 buffer; then bead-associated proteins were denatured by boiling in loading buffer at 100 °C for 10 min.

### Antibodies and reagents

Antibodies and other reagents were obtained as follows: anti-AF6 and anti-RIPK1(receptor-interacting serine/threonine kinase 1) antibodies from BD Transduction Laboratories; anti-pRIPK1(S166), anti-TNFR1(TNF receptor superfamily member 1A), anti-Hsp90(heat shock protein 90), and anti-GAPDH(glyceraldehyde-3-phosphate dehydrogenase) antibodies from Cell Signaling Technology; anti-pRIPK3(Ser232) (receptor-interacting serine/threonine kinase 3), anti-pMLKL(Ser345)(mixed lineage kinase domain-like pseudokinase), anti-MLKL and anti-CYLD(CYLD lysine 63 deubiquitinase) antibodies from abcam; anti-USP21, anti-FADD (Fas-associated via death domain), anti-TRAF2(TNF receptor-associated factor 2), and anti-TRADD(TNFRSF1A associated via death domain) antibodies from Santa Cruz Biotechnology; anti-A20(TNF alpha-induced protein 3) antibody from ABClonal; anti-RIPK3 from Prosci; and anti-actin antibody from Servicebio. Smac was a gift from Haibing Zhang’s lab. Other reagents were purchased as follows: mouse TNFα (Minneapolis), lipopolysaccharide (LPS) (Sigma-Aldrich Cat. L4516), Nec-1 (Enzo Life Science), Z-Val-Ala-Asp (OMe)-FMK (z-VAD) and Bv6 (MedChemExpress).

### Cell lines

Mouse embryonic fibroblasts (MEFs), L929 (a normal murine fibroblast cell line), BMDMs and 293 T (a human kidney-derived epithelial-like cell line) were cultured in high-glucose DMEM (Gibco) or RIPA-1640 (Life Technologies) medium containing 10% fetal bovine serum (Hyclone) and 1% penicillin/streptomycin (Life Technologies) under 5% CO_2_ at 37 °C. Transfection of plasmids and small interfering RNAs into cells was performed using Lipofectamine 3000 reagent (Life Technologies) according to the manufacturer’s protocol.

Primary MEFs were isolated from E11.5 littermate embryos. After dissecting and removing the head and visceral tissues, remaining tissues were incubated with 5 mL 0.25% trypsin/EDTA solution (Gibco) at 37 °C for 30 min. Following digestion, DMEM medium (5 mL) was added to the tissue to terminate digestion. The suspension was centrifuged at 900 *g* for 5 min, and the MEFs then were resuspended in DMEM medium supplemented with 10% FBS and 1% penicillin/streptomycin spread on cell culture plates and grown at 37 °C with 5% CO_2_.

Prior to the isolation of BMDMs, L929 cells were cultured to obtain a culture supernatant containing macrophage colony-stimulating factor. Primary BMDMs were obtained from adult mice. After animals were euthanized and disinfected with alcohol, the tibia and femur were exposed and cut; the bone marrow was flushed from the bones with a syringe containing phosphate-buffered saline (PBS). The resulting suspension was centrifuged at 1300 *g* for 5 min, then resuspended in a red blood cell lysate and incubated at room temperature for 5 min. The suspension again was centrifuged at 1300 *g* for 5 min, and the cells were then resuspended in medium (69% 1640 medium, 20% L929 culture supernatant, 10% inactivated fetal bovine serum and 1% penicillin/streptomycin)(s), spread on 10-cm cell culture plates and grown at 37 °C with 5% CO_2_.

### Stable cell-line construction

Plasmids construction of pLKO.1 and pLKO.1-shAF6 [16], lentivirus production, and stable cell-line constructions [17] were described previously. The virus particles were produced by transient transfection into 293 T cells seeded in 10-cm dishes with 2.7 mg of the packaging construct, 0.9 mg of the construct that expresses the vesicular stomatitis virus glycoprotein and 2.7 mg of the pLKO.1 or pLKO.1-shAF6 plasmids in DMEM medium (containing 20% fetal bovine serum and 1% penicillin/streptomycin), and the media from the transfected cells was collected as the cells were re-fed each day for 3 days. The virus-containing media was pooled, 0.22 μm filtered, and used to generate L929 AF6-knock down stable cell lines.

### Cell survival assay

Cell survival was assessed using the Cell Titer-Glo Luminescent Cell Viability Assay kit (Promega Cat. G7572) and the luminescence was measured with an Empire microplate reader.

### Administration of recombinant adenoviruses

The AF6-overexpressing adenovirus was purchased from Hanbio. The adenovirus stock was diluted in PBS and administered at a dose of 1$\times$10^7^ plaque-forming units (PFU)/well to cells and by intravenous injection (via a lateral tail vein) at 1$\times$10^9^ PFU/mouse in mice. We confirmed that injection of the AF6-overexpressing adenoviruses did not affect food consumption compared with that of control (PBS-treated) animals (data not shown).

### RNA-seq and data analysis

For RNA-seq, total RNA was collected from isolated liver tissues and extracted by Trizol. The quality of RNA samples and cDNA libraries were constructed and sequenced by Majorbio Biotech. GESA and cluster analysis were performed by Majorbio Biotech.

### Statistical analysis

All in vitro experiments were performed at least three times, and all in vivo experiments were performed at least twice. Pairwise comparisons between groups were conducted using two-tailed non-paired Student’s t-tests. *p* values < 0.05 were considered statistically significant. In figures, differences are indicated as *p < 0.05, **p < 0.01, ***p < 0.001, or not significant (ns; *p* ≥ 0.05). Statistical analyses were conducted using Graphpad Prism 8.0.

## Results

### AF6 expression and necroptosis are upregulated and correlate with the progression of NASH

**Fig. 1:**
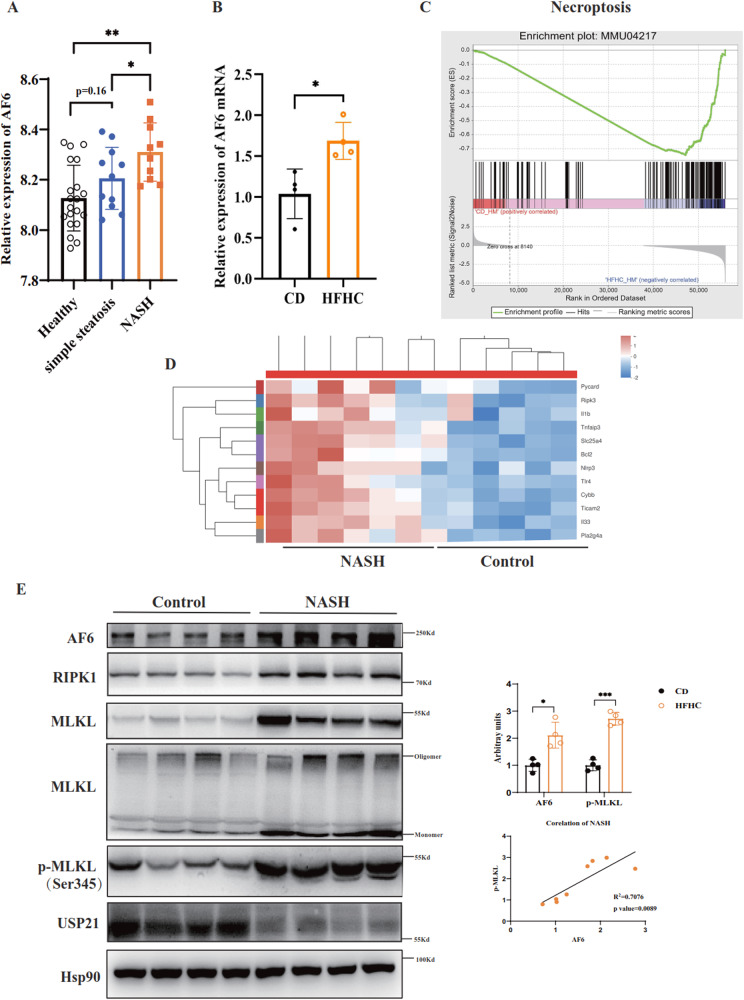
AF6 expression and necroptosis are up-regulated and correlate with the progression of NASH. **A** Hepatic levels of AF6 in healthy patients (*n* = 20) and in patients with simple steatosis (*n* = 11) and non-alcoholic steatohepatitis (NASH) (*n* = 10) in GSE89632. **B** mRNA levels of *Af6* in mice maintained on a high-fat and high-cholesterol (HFHC) diet for 16 weeks. **C** Gene set enrichment analysis (GSEA) and **D** heatmap of necroptosis in HFHC model. **E** Immunoblot of AF6, RIPK1, MLKL, MLKL oligomer, p-MLKL, USP21, and Hsp90 in liver after maintenance on HFHC diet. Densitometric analysis was used to quantify band intensities; the protein levels of AF6 and p-MLKL were normalized to that of the housekeeping protein Hsp90 in the respective lanes. Correlation between AF6 and p-MLKL levels was assessed using Graphpad Prism 8.0. Data are expressed as mean ± SEM. Pairwise comparisons between groups were conducted using two-tailed non-paired Student’s *t* tests. ns, not significant (*p* ≥ 0.05); *p < 0.05; **p < 0.01; ***p < 0.001.

Non-alcoholic fatty liver disease (NAFLD), which is related to obesity, inflammation, and metabolic syndrome, has become the world’s most widespread liver disease today, representing an epidemic that is a serious threat to global health [18]. Furthermore, NAFLD can evolve into NASH, fibrosis, cirrhosis and even hepatocellular carcinoma in severe cases. There is a major ongoing research effort to identify the drivers of NAFLD, a condition that may correlate with systemic metabolic disease. In previous studies, we showed that the hepatic polar protein AF6 directly regulates blood glucose homeostasis and insulin sensitivity in two murine diabetes models, specifically HFD and *db/db* mice [9]. That work unequivocally demonstrated that AF6 is up-regulated under pathological conditions of liver fat accumulation [9]. Consistent with those results, we observed marked and dose-dependent accumulation of AF6 in primary hepatic epithelial cells following exposure to OA or PA (Supplementary Fig. 1a). We further investigated the AF6 expression in clinical patients with NASH. As we saw in patients with diabetes, the accumulation of AF6 increased in patients with NAFLD in several databases, and the levels of AF6 appeared to gradually increase with NASH progression (Fig. 1a and Supplemental Fig. 1b). To gain more insight into the role of AF6 in in vivo pathogenicity, we established a NASH model by maintaining mice on a HFHC diet for 16 weeks and AF6 levels (at both the mRNA and protein levels) were elevated in the mouse HFHC model (Fig. 1b, e).

Continued damage and inflammation of the liver can cause abnormal death of hepatocyte, leading to a sharp decline in liver function. Recent studies have also shown that necroptosis, attributable to inflammation and disease development, may contribute to the progression of liver disease and metabolic dysfunction [19–23]. Therefore, RNA sequencing (RNA-seq) experiments were performed to evaluate the nature of cell death in mice experiencing NASH. Indeed, GESA and cluster analysis of RNA-seq data revealed considerable enrichment for transcripts of genes associated with necroptosis following the induction of NASH by HFHC feeding (Fig. 1c and d). Similarly, transcripts of genes associated with TNF$\alpha$ signaling, a pathway that contributes to cell death and inflammation [24, 25] were also enriched in this mouse model of NASH (Supplementary Fig. 1c). While most targets of NF-κB signaling which promotes cell survival and involving in the maintenance of inflammatory homeostasis were decreased in GSE89632 (Supplementary Fig. 1d). The mouse HFHC model demonstrated accumulation of AF6 and the phosphorylated version of the pseudokinase mixed lineage kinase domain-like protein (p-MLKL) (Fig. 1e), a primary effector and marker of necroptosis [26, 27]. We also found that the expression of MLKL oligomers and RIPK1, an upstream kinase of necroptosis, were also increased. Notably, the levels of AF6 and p-MLKL exhibited a strong correlation (*R*^2^, 0.7076; *p* < 0.01) in NASH mice (Fig. 1e). Together, these results suggested that AF6 levels affect not only insulin resistance but also necroptosis-related hepatic damage.

### AF6 exacerbates necroptosis and cell death in vitro

**Fig. 2:**
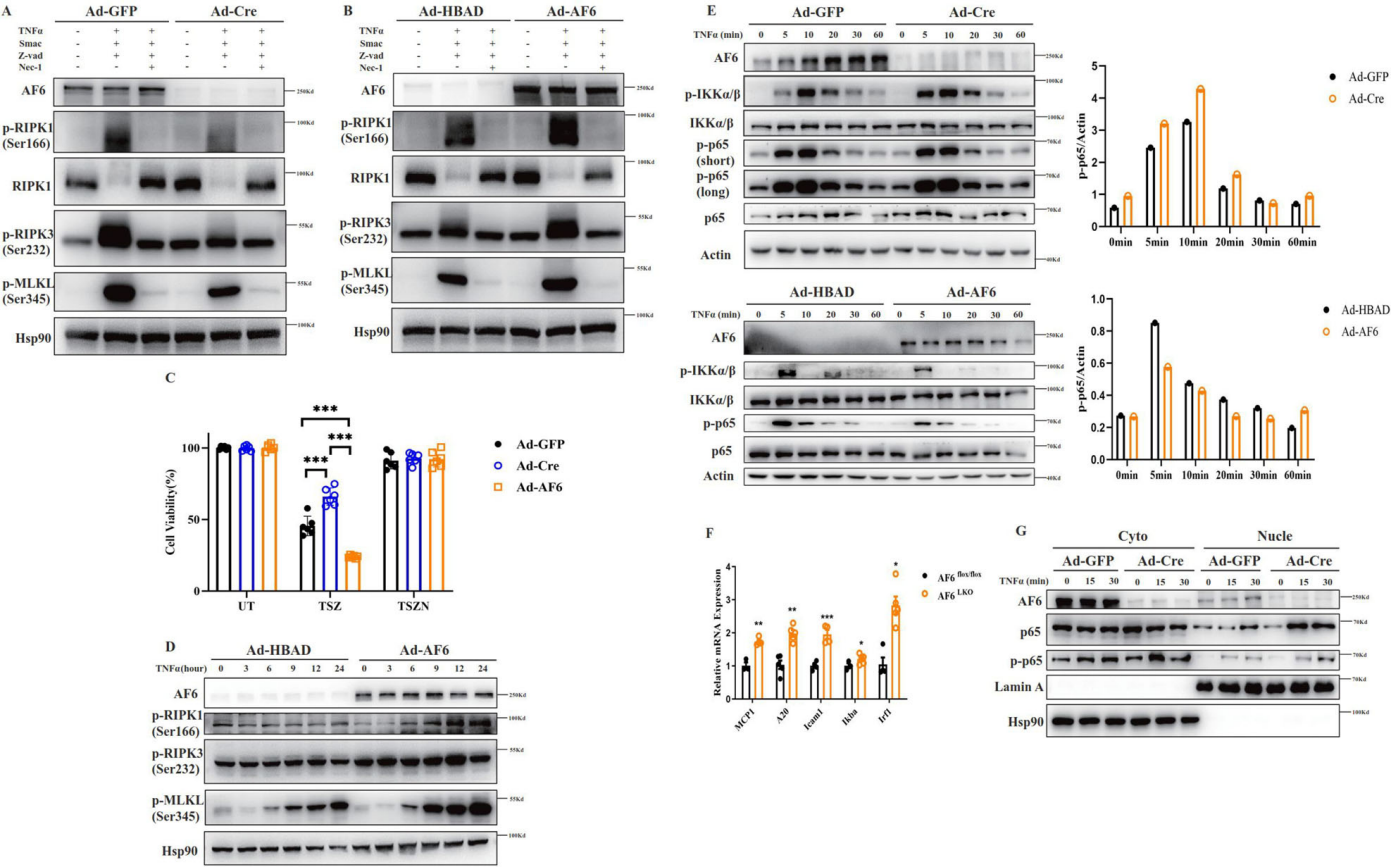
AF6 exacerbates necroptosis and cell death in vitro. The levels of key molecules of the necroptosis pathway were determined by immunoblotting of *Af6*^*flox/flox*^ MEF cells infected with adenoviruses encoding green fluorescent protein (Ad-GFP) or Cre recombinase (Ad-Cre) (**A**), or with empty adenovirus vector (harboring the *HBAD* promoter alone; Ad-HBAD) or adenovirus encoding the polarity protein AF6 (Ad-AF6) (**B**). Following infection cells were grown (as indicated) in the absence (−) or presence (+) of tumor necrosis factor (TNF) α (cytokine; 50 ng/mL), Smac mimetic (inhibitor of inducers of apoptotic proteins (IAPs); 100 nM), z-VAD (pan-caspase inhibitor, inducer of necroptosis; 20 μM) and Nec-1 (inhibitor of RIPK1; 30 μM). **C** Cell viability was assessed (using the CellTiter-Glo kit) in *Af6*^*flox/flox*^ MEF cells infected with Ad-GFP, Ad-Cre or Ad-AF6 and then grown in the presence of media containing vehicle (untreated; UT), TNFα, Smac mimetic and z-VAD (TSZ), or TNFα, Smac mimetic, z-VAD and Nec-1 (TSZN). **D** L929 cells infected with Ad-HBAD or Ad-AF6 were grown in the presence of TNFα (50 ng/mL) and sampled at the indicated time points (in hours); lysates of the samples were analyzed by immunoblotting using antibodies against the indicated proteins. **E** Primary hepatocytes of *Af6* ^*flox/flox*^ (the parent strain; produces AF6), *Af6*^*LKO*^ (liver-specific deletion of the AF6-encoding locus) and primary hepatocytes of *Af6* ^*flox/flox*^ after Ad-HBAD (AF6 normal expression) and Ad-AF6 (AF6 overexpression) adenovirus infection were grown in the presence of TNFα (50 ng/mL) and sampled at the indicated time points (in hours); lysates of the samples were analyzed by immunoblotting using antibodies against the indicated proteins of the NF-κB signaling pathway. **F** Primary hepatocytes of *Af6*^*flox/flox*^ and *Af6*^*LKO*^ were grown in the presence of TNFα (50 ng/mL) for 90 min, and levels of transcripts encoding NF-κB-targeted genes were determined by reverse transcription-polymerase chain reaction (RT-PCR. Values were normalized to the mRNA level of the housekeeping gene 36b4 in the respective sample. *Mcp1*, encoding monocyte chemoattractant protein-1. *A20*, encoding the A20 deubiquitinase. *Icam1*, encoding intracellular adhesion molecule 1. *Ikba*, encoding IκB. Irf1, encoding interferon regulatory factor 1. **G** Primary hepatocytes infected with Ad-GFP or Ad-Cre were grown in the presence of TNFα (50 ng/mL) and sampled at the indicated times (in minutes); cell lysates were then separated into cytoplasmic (Cyto) and nuclear (Nucle) fractions, and analyzed by immunoblotting using antibodies against AF6, p65 (a component of NF-κB known to translocate to the nucleus), p-p65, Lamin A (a nuclear marker), and Hsp90 (heat shock protein 90; a cytoplasmic marker). Data are expressed as mean ± SEM. Pairwise comparisons between groups were conducted using two-tailed non-paired Student’s *t* tests. ns, not significant (*p* ≥ 0.05); *p < 0.05; **p < 0.01; ***p < 0.001. Band sizes (in kD; based on size ladders (not shown)) are indicated to the right of each immunoblot strip.

To further examine the role of AF6 in necroptosis, we isolated MEF cells, which are known to be sensitive to necroptosis and in which necroptosis can be induced in vitro. The phosphorylation levels of RIPK1, RIPK3, and MLKL, the key downstream kinase regulators of necroptosis, were significantly attenuated when AF6 levels were knocked down in MEF cells, while overproduction of AF6 resulted in significant potentiation of the phosphorylated versions of these proteins (Fig. 2a, b). Similar results were obtained in primary hepatocytes, L929 cells and BMDMs (Supplemental Fig. 2a, b, d and f). To evaluate the impact of AF6 levels on TNFα-associated cell survival, we assessed the effect of AF6 level on cell survival following exposure to a combination of reagents (TNFα, a Smac mimetic (an inhibitor of inducers of apoptotic proteins (IAPs) and z-VAD (a pan-caspase inhibitor)) known to induce necroptosis. Compared to untreated (UT) cells, AF6 knockdown (KD) cells underwent significantly less cell death upon growth in the presence of these agents (TSZ). In contrast, AF6-overproducing cells grown in the presence of this agent showed increased mortality compared to control cells (Fig. 2c). The potentiation of necroptosis signaling and mortality by AF6 was completely blocked by the addition of Nec-1(TSZN), an inhibitor of RIPK1 kinase activity (Fig. 2a–c). Similar results were obtained in primary hepatocytes, L929 cells and BMDMs (Supplemental Fig. 2c, e, and g). In contrast, AF6-overproducing L929 cells grown in the present of TNFα stimulation exhibited readily detected levels of p-RIPK1 (Ser 166), p-RIPK3 (Ser 232), and p-MLKL (Ser 345), factors known to be associated with necroptosis (Fig. 2d), again suggesting that AF6 potentiates necroptosis.

Previous reports identified the ubiquitination of RIPK1 as a key factor in determining cell fate, and ubiquitination of RIPK1 activates the nuclear factor (NF) -$\kappa$B signaling pathway, leading to increased cell survival and inflammation in liver disease [28]. Therefore, we next assessed the effect of AF6 levels on NF- $\kappa$B signaling. First, we demonstrated that AF6 KD in primary hepatocytes resulted in significant activation of the NF- $\kappa$B signaling pathway (compared to hepatocytes with normal AF6 expression), as assessed by the expression of the gene targets of this pathway and changes in cell survival following growth in the presence of TNFα (Fig. 2e, f). Meanwhile, overexpression of AF6 inhibited activation of NF- $\kappa$B signaling (Fig. 2e). Consistent with those results, TNFα-stimulated AF6 KD primary hepatocytes demonstrated significant increases in the nuclear localization of p65 (a component of NF-κB known to translocate to the nucleus) and p-p65 compared to hepatocytes with normal AF6 expression (Fig. 2g). Considered together, these data indicated that elevated expression of AF6 renders hepatic cells more sensitive to TNFα-induced necroptosis and hepatocyte death. Mechanistically, this process may be associated with the ubiquitination of RIPK1 and activation of the NF-$\kappa$B signaling pathway.

### AF6 interacts with RIPK1 via changes in ubiquitination of K376

**Fig. 3:**
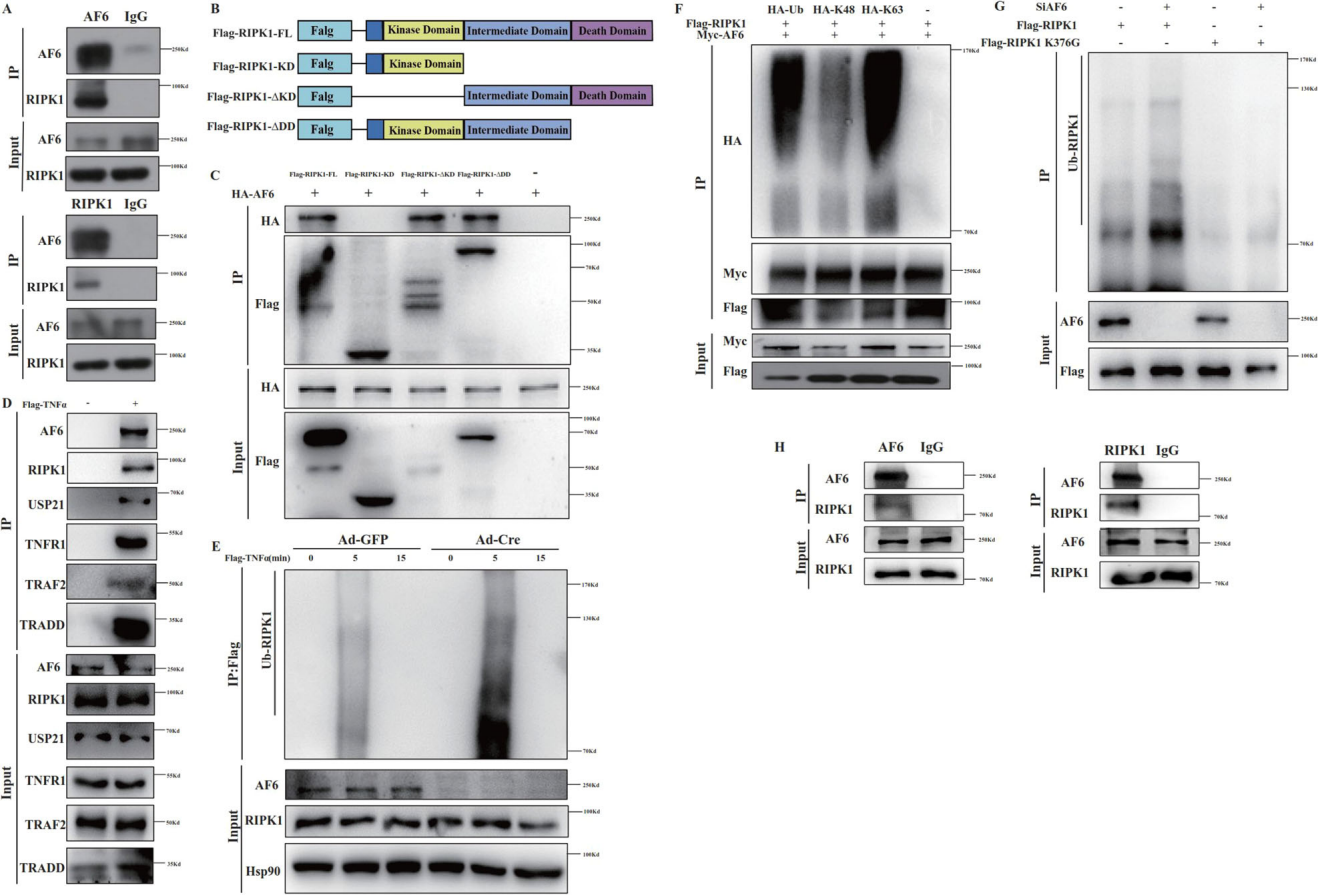
AF6 can interact with RIPK1 and impact its ubiquitination. **A** Endogenous AF6 bound to endogenous RIPK1 in *Af6*^*flox/flox*^ mice primary hepatocytes. Cells were lysed with NP-40 buffer and incubated with control mouse IgG or anti-AF6, anti-RIPK1 monoclonal antibody for 4 h, then immunoprecipitated with A/G-agarose beads for 2 h at room temperature and lysates of the samples were analyzed by immunoblotting using antibodies against the indicated proteins. **B** Plasmids construction model of different domains of RIPK1. **C** 293T cells were transfected with expression vectors for HA-tagged AF6 and Flag-tagged full-length, ∆KD, KD, and ∆DD RIPK1 for 24 h, cells were lysed with NP-40 buffer and then immunoprecipitated with anti-FLAG beads for 4 h at room temperature and immunoblotted as indicated using antibodies against the indicated proteins. **D** Primary hepatocytes of *Af6*^*flox/flox*^ mice were stimulated with Flag- TNFα (100 ng/mL) for 5 min and lysed with NP-40 buffer and immunoprecipitated with anti-FLAG Beads for 4 h at room temperature and immunoblotted as indicated using antibodies against the indicated proteins. **E** The ubiquitination of RIPK1 in primary hepatocytes of *Af6*^*flox/flox*^ mice after Ad-GFP and Ad-Cre adenovirus infection was detected by immunoprecipitation with anti-FLAG Beads for 4 h at room temperature after treating with Flag-TNFα (100 ng/mL) for indicated time and immunoblotted as indicated using antibodies against the indicated proteins. **F** 293 T cells were transfected with expression vectors for HA-tagged Ub, K48, K63 ubiquitin, Flag-tagged RIPK1, and Myc-tagged AF6 for 24 h and lysed with NP-40 buffer, then immunoprecipitated with anti-RIPK1 monoclonal antibody and A/G-agarose beads for 4 h at room temperature and immunoblotted as indicated using antibodies against the indicated proteins. **G** 293 T cells were transfected with expression vectors for Flag-tagged WT and K376G RIPK1 with or without SiAF6 transfection for 24 h. All cells were lysed with NP-40 buffer, immunoprecipitated with anti-FLAG beads for 4 h at room temperature, and immunoblotted as indicated using antibodies against the indicated proteins. **H** Endogenous AF6 bound to endogenous RIPK1 in RIPK1^K376R/ K376R^ MEF cells. Cells were lysed with NP-40 buffer and incubated with control mouse IgG or anti-AF6, anti-RIPK1 monoclonal antibody for 4 h, then immunoprecipitated with A/G-agarose beads for 2 h at room temperature and lysates of the samples were analyzed by immunoblotting using antibodies against the indicated proteins.

Our demonstration that AF6 contributes to necroptosis motivated us to seek the underlying mechanism(s) of this process. Given the paramount role of RIPK1 and its ubiquitination state in deciding cell fate, we next assessed whether AF6 might interact physically with RIPK1. In primary hepatocytes and MEFs, AF6 and RIPK1 showed an endogenous interaction, as shown by co-immunoprecipitation (Fig. 3a and Supplementary Fig. 3a). RIPK1 contains three domains that contribute to the protein’s function: a N-terminal kinase domain (KD), an intermediate domain and a C-terminal death domain (DD). We constructed plasmids encoding FLAG-tagged version of RIPK1 proteins (including tagged full-length RIPK1 (FLAG-RIPK1), tagged KD alone (FLAG-KD), tagged RIPK1 lacking the KD (FLAG-∆KD) and tagged RIPK1 lacking the DD (FLAG-${{\Delta }}$DD)) (Fig. 3b) and transfected these constructs into 293T cells; the resulting transfectants were subjected to immunoprecipitation to determine the roles of various RIPK1 domains in the protein’s interactions with AF6. The results showed that AF6 bound to FLAG-RIPK1, FLAG-${{\Delta }}$KD and FLAG-${{\Delta }}$DD, but not to FLAG-KD, indicating that AF6 bound interacts with RIPK1 via the intermediate domain (Fig. 3c).

Previous work has shown that a protein assembly, termed Complex I, is formed by TNF receptors following stimulation with the cytokine; forming this assembly is considered the first step in the cells survival or death during disease progression [29, 30]. We postulated that AF6 is recruited into the TNFα receptor Complex I. Indeed, in primary hepatocytes and MEFs were grown in the presence of FLAG-TNFα, immunoprecipitation with anti-FLAG tag beads pulled down AF6, confirming the existence of an endogenous interaction between AF6 and RIPK1 and the incorporation of AF6 into Complex I (Fig. 3d and Supplementary Fig. 3b). Next, we tested the effect of AF6 on the ubiquitination of RIPK1. Firstly, we showed that hepatocyte-specific knockout of AF6 resulted in increased ubiquitination of RIPK1 (Fig. 3e). Secondly, AF6 KD in MEFs significantly elevated the ubiquitination of RIPK1; while overexpression of AF6 led to decreased RIPK1 ubiquitination (Supplemental Fig. 3c). Previous studies have found that the K376R mutation of RIPK1 results in embryogenic lethality [31], with the livers of the affected embryos exhibiting high levels of cell death and severe inflammation. K11-, K48-, K63-, and Met1-linked ubiquitination of RIPK1 has been shown regulate its function transition [32] and different E3 ligase-involved ubiquitination of RIPK1 emerged as a pivotal player in preventing pathological outcomes of RIPK1 signaling. Given that AF6 interacts with the intermediate domain of RIPK1, and the K63-linked ubiquitination of K376 is closely linked to the formation of Complex I and the function of RIPK1 [33, 34], we assessed the role of RIPK1 residue 376 by constructing a plasmid that encodes FLAG-tagged RIPK1 with a K376G substitution. Firstly, we confirmed that AF6 mainly affected K63-linked ubiquitination of RIPK1 by co-transfection of AF6, RIPK1 and different ubiquitin (Ub) plasmids and immunoprecipitation (Fig. 3f). Secondly, we showed that the enhance of RIPK1 ubiquitination seen with AF6 KD, and this phenomenon was blocked after mutation of RIPK1 376 site (Fig. 3g). To confirm whether 376 site of RIPK1 was required for the interaction between AF6 and RIPK1, co-immunoprecipitation assay was performed in MEF cells harboring RIPK1 376 site mutation(Fig. 3h). We found that the interaction between AF6 and RIPK1 was not affected by the mutation at 376 site, and AF6 mainly by impairing the degree of ubiquitination at 376 site of RIPK1. Together, we show that changes in the ubiquitination of RIPK1 on K376 serve distinct regulatory functions in AF6-dependent necroptosis.

### AF6 alters RIPK1 ubiquitination through deubiquitylation

**Fig. 4:**
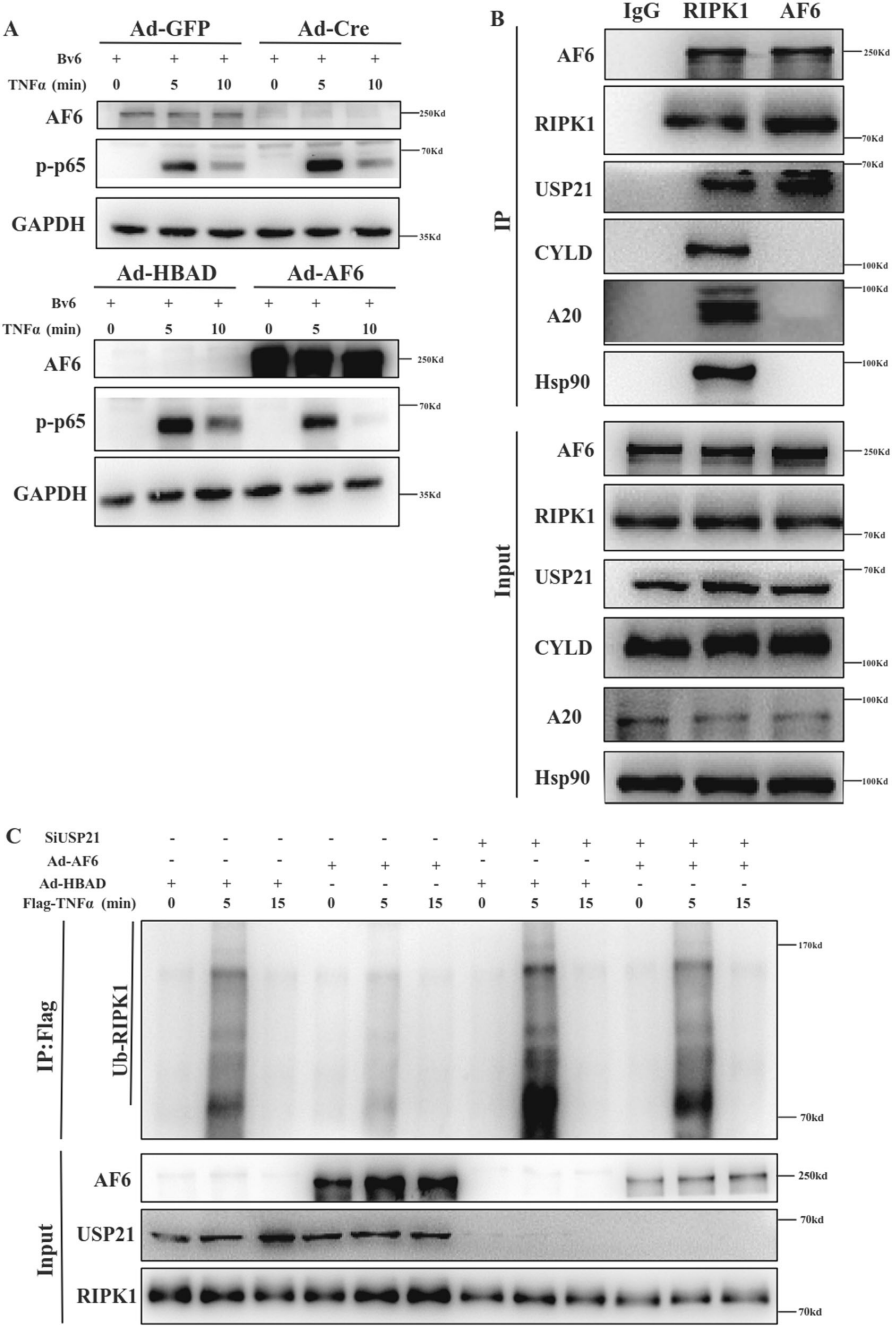
AF6 affects RIPK1 ubiquitination through deubiquitylase enzyme USP21. **A** Primary hepatocytes of *Af6*^*flox/flox*^ mice were grown in the presence of media containing TNF α (50 ng/mL) and BV6 (5 μM) for indicated time (in minutes), then lysed with NP-40 buffer and immunoblotted as indicated using antibodies against the indicated proteins. **B** Primary hepatocytes of *Af6*^*flox/flox*^ mice were lysed with NP-40 buffer, immunoprecipitated with anti-IgG, RIPK1, and AF6 monoclonal antibodies and A/G-agarose beads for 4 hours at room temperature and immunoblotted as indicated using antibodies against the indicated proteins. **C** *Af6*^*flox/flox*^ MEF cells were infected with Ad-HBAD and Ad-AF6 adenovirus and transfected with SiNC and SiUSP21 for 24 h. MEF cells were stimulated with Flag-TNFα (100 ng/mL) for indicated time. Then, cells were lysed with NP-40 buffer, immunoprecipitated with anti-FLAG beads for 4 h at room temperature, and immunoblotted as indicated using antibodies against the indicated proteins.

The preceding experiments revealed that AF6 affects the occurrence of necroptosis, but we also observed that pre-treatment of cells with the Cellular inhibitor of apoptosis protein (cIAP) and the X-linked inhibitor of apoptosis protein did not block AF6’s role in the activation of NF-$\kappa$B signaling (Fig. 4a). To clarify the mechanism whereby AF6 modulates RIPK1 ubiquitination, we tested the possible interaction of AF6 with several known deubiquitinating enzymes (including cylindromatosis (CYLD), A20 (also known as TNFα-induced protein 3) and heat shock protein 90 (Hsp90)) that have been reported to affect RIPK1 ubiquitination. Immunoprecipitation failed to detect any physical interaction between AF6 and any of those deubiquitylases (Fig. 4b). However, querying of the (BioGRID) [35] database showed that USP21 interacts with AF6 and USP21 regulates the K63-linked ubiquitination of RIPK1 and activation of NF-κB signaling following stimulation with TNFα [36]. Using immunoprecipitation, we demonstrated that AF6, RIPK1, and USP21 interacted with each other, forming a protein complex in lysates of primary hepatocytes (Fig. 4b). Next, we showed that overexpression of AF6 abrogated RIPK1 ubiquitination in MEFs upon TNF stimulation, and further knockdown of USP21 levels rescued the reduction in RIPK1 ubiquitination caused by AF6 overexpression (Fig. 4c). These data implied that hepatocyte-intrinsic AF6 triggers the ubiquitination of RIPK1; thus, AF6 alters RIPK1 deubiquitination depends on the regulation of USP21 activity.

### In vivo overexpression of AF6 increases animal sensitivity to TNFα-induced SIRS

**Fig. 5:**
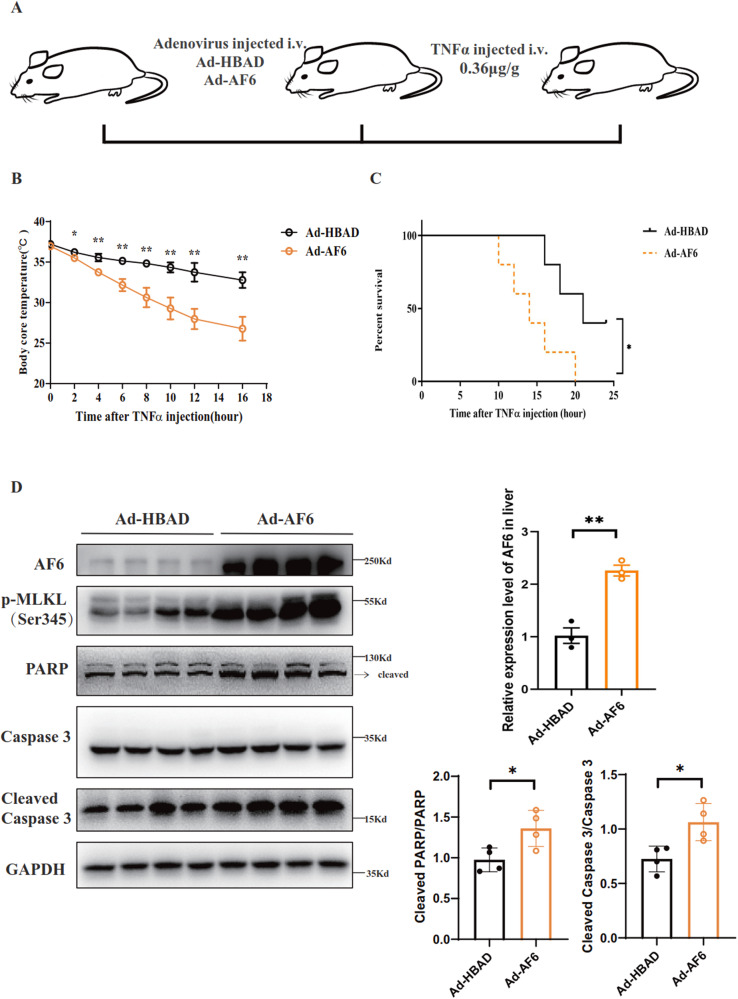
Animal that over-expressing AF6 is sensitive to TNFα−induced SIRS in vivo. **A** Work model of TNF (360 μg/kg) induced SIRS. Core temperature (**B**) were measured every hour after TNFα injection and survival (**C**) of mice was recorded with or without AF6 overexpression. **D** Immunoblot of proteins relating to cell death signaling pathway in liver after TNFα injection and the effective efficiency of hepatic *Af6* overexpression at mRNA levels was examined by RT-PCR. Densitometric analysis was used to quantify band intensities; the cleaved-PARP and cleaved-Caspase3 protein levels were normalized to that of the housekeeping protein GAPDH in the respective lanes. Data are expressed as mean ± SEM. Pairwise comparisons between groups were conducted using two-tailed non-paired Student’s t-tests. ns not significant (*p* ≥ 0.05); *p < 0.05; **p < 0.01; ***p < 0.001.

Elevated TNFα levels are known to be clinically associated with Systemic Inflammatory Response Syndrome (SIRS), leading to progression of hepatic encephalopathy, renal failure, and poor outcomes in patients with cirrhosis [37, 38]. Similar effects are seen in mice administered systemically with TNFα, leading to the use of TNFα-injected mice as an animal disease model for the study of cell death and cytokine responses [39, 40]. To further elucidate the role of AF6 in SIRS, we infected C57BL/6 mice with Ad-AF6 to provide in vivo AF6 overexpression, and then injected these animals IV with TNFα (Fig. 5a). Mice overexpressing AF6 showed elevated sensitivity to SIRS, as demonstrated by lower core temperatures, increased mortality, and earlier death compared to wild-type mice (Fig. 5b, c).

Next, we tested the liver expression of cell death-relevant proteins in AF6-overexpressing mice in SIRS model. The fold change of AF6 in the liver was first examined (Fig. 5d).AF6 overexpression was associated with accumulation (compared to mice lacking AF6 overexpression) in the liver of p-MLKL, cleaved PARP (poly (ADP-ribose) polymerase 1), and cleaved caspase 3, all of which are cell death effectors (Fig. 5d). Similar accumulation was detected in the pancreas of the AF6-overexpressing animals in SIRS model, along with a larger area of necrosis in the pancreas (Supplementary Fig. 4a, b). Thus, the increased sensitivity to SIRS of AF6-overexpressing mice appears to reflect increased apoptosis and activation of necroptosis in response to TNFα stimulation.

### Murine liver knockout of *Af6* protects cells from necroptosis and additional consequences in various liver diseases

**Fig. 6:**
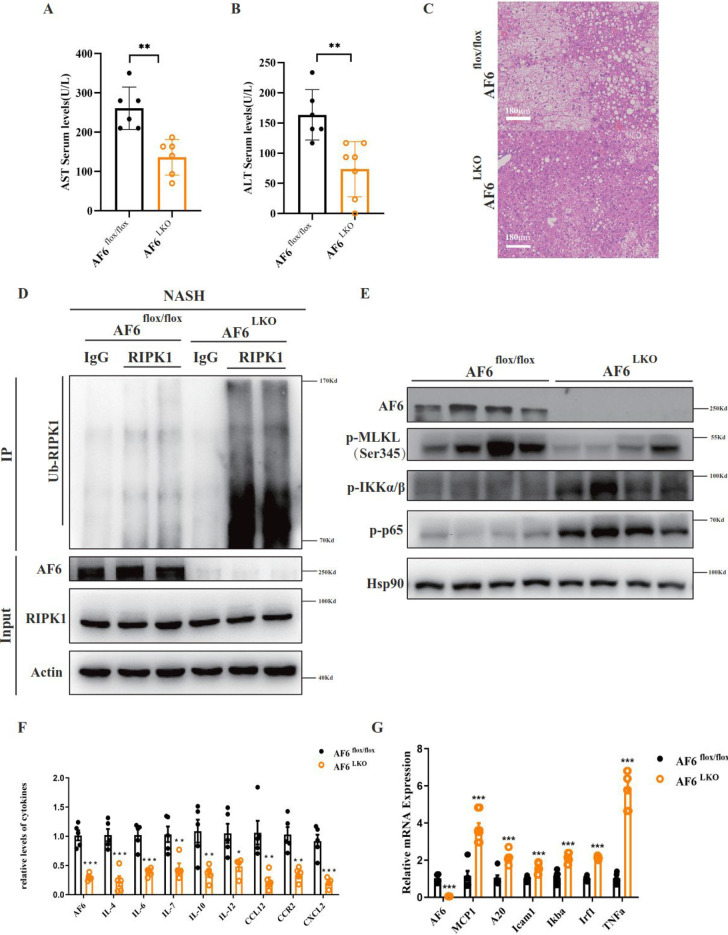
Liver knockout of AF6 can slow the progression of NASH through necroptosis. The circulating levels of serum AST (**A**) and ALT (**B**) were measured after HFHC maintenance in *Af6*^*flox/flox*^ and *Af6*^*LKO*^ mice by kit. **C** HE staining of pathological sections in *Af6*^*flox/flox*^ and *Af6*^*LKO*^ mice after HFHC feeding. **D** The liver tissues from *Af6*^*flox/flox*^ and *Af6*^*LKO*^ mice were lysed with NP-40 buffer, immunoprecipitated with anti-RIPK1 monoclonal antibodies and A/G-agarose beads for 4 h at room temperature. The ubiquitination of RIPK1 was immunoblotted as indicated using antibodies against the indicated proteins. **E** Immunoblot of proteins relating to necroptosis and NF-κB signaling pathway in liver tissues in *Af6*^*flox/flox*^ and *Af6*^*LKO*^ mice after HFHC diet maintenance. mRNA transcription changes of (**F**) inflammatory cytokines genes and (**G**) NF-κB targeted genes were determined by quantitative RT-PCR. Values were normalized to the mRNA level of the housekeeping gene *36b4* in the respective sample. *IL4*, encoding interleukin 4. *IL6*, encoding interleukin 6. *IL7*, encoding interleukin 7. *IL10*, encoding interleukin 10. *IL12*, encoding interleukin 12. *CCL12*, encoding chemokine (C-C motif) ligand 12. *CCR2*, encoding C-C motif chemokine receptor 2. *CXCL2*, encoding C-X-C motif chemokine ligand 2. Data are expressed as mean ± SEM. Pairwise comparisons between groups were conducted using two-tailed non-paired Student’s *t* tests. ns, not significant (*p* ≥ 0.05); *p < 0.05; **p < 0.01; ***p < 0.001.

Given our demonstration that AF6 affects the regulation of both cell death and pro-inflammatory signaling pathways, we assessed these effects in other mouse models of liver diseases, including those for acute liver injury, liver fibrosis and diabetes. Notably, we obtained results similar to those seen in our mouse model of NASH. Specifically, we performed the analysis in mice engineered to have liver-specific elevation of AF6 expression (Supplementary Fig. 5a–c), suggesting that AF6 modulates the occurrence of necroptosis in these additional liver diseases. Next, we generated mice with liver-specific knockout (LKO) of the *Af6* gene. Specifically, exon 2 of the *Af6* locus in these mice was flanked by *flox* sites; upon mating of these *Af6*^*flox/flox*^ mice (which show normal AF6 expression) to animals harboring a construct providing liver-specific expression of the Cre recombinase, *Af6* was efficient deleted in a liver-specific manner (*Af6*^*LKO*^), as confirmed Supplemental Fig. 5d. Among mice maintained on the HFHC diet, the NASH-associated severe hepatocyte damage and extensive liver necrosis seen in *Af6*^*flox/flox*^ were ameliorated in *Af6*^*LKO*^ mice (Fig. 6c). Consistent with these observations, these HFHC-fed *Af6*^*LKO*^ mice exhibited decreased serum levels of Asp aminotranferase (AST) and Ala aminotransferase (ALT), as well as a decline in liver fat accumulation and inflammation, compared to NASH *Af6*^*flox/flox*^ animals (Fig. 6a–c). In confirmation of our earlier results, liver RIPK1 was more strongly ubiquitinated in the NASH *Af6*^*LKO*^ mice than in the NASH *Af6*^*flox/flox*^ animals (Fig. 6d), while signaling via the necroptotic pathway in liver tissue was decreased, as evidence by decreased p-MLKL levels (Fig. 6e). At the same time, the livers of the NASH *Af6*^*LKO*^ showed activation of the NF-$\kappa$B signaling pathway, as evidenced by increased expression of NF-$\kappa$B target genes, implying increases in cell survival and the inflammatory response (Fig. 6f, g). These results demonstrated that hepatic knockout of *Af6* is sufficient to protect liver from NASH-associated injury and inflammation, and that these effects are mediated by increased levels of RIPK1 ubiquitination resulting in decreased necroptosis. In contrast, abnormal accumulation of AF6 halts the inhibition of necroptosis and is tightly associated with the lethal pathology of hepatocyte death liver.

### MLKL mediates AF6-activation of necroptosis in response to acute liver injury

**Fig. 7:**
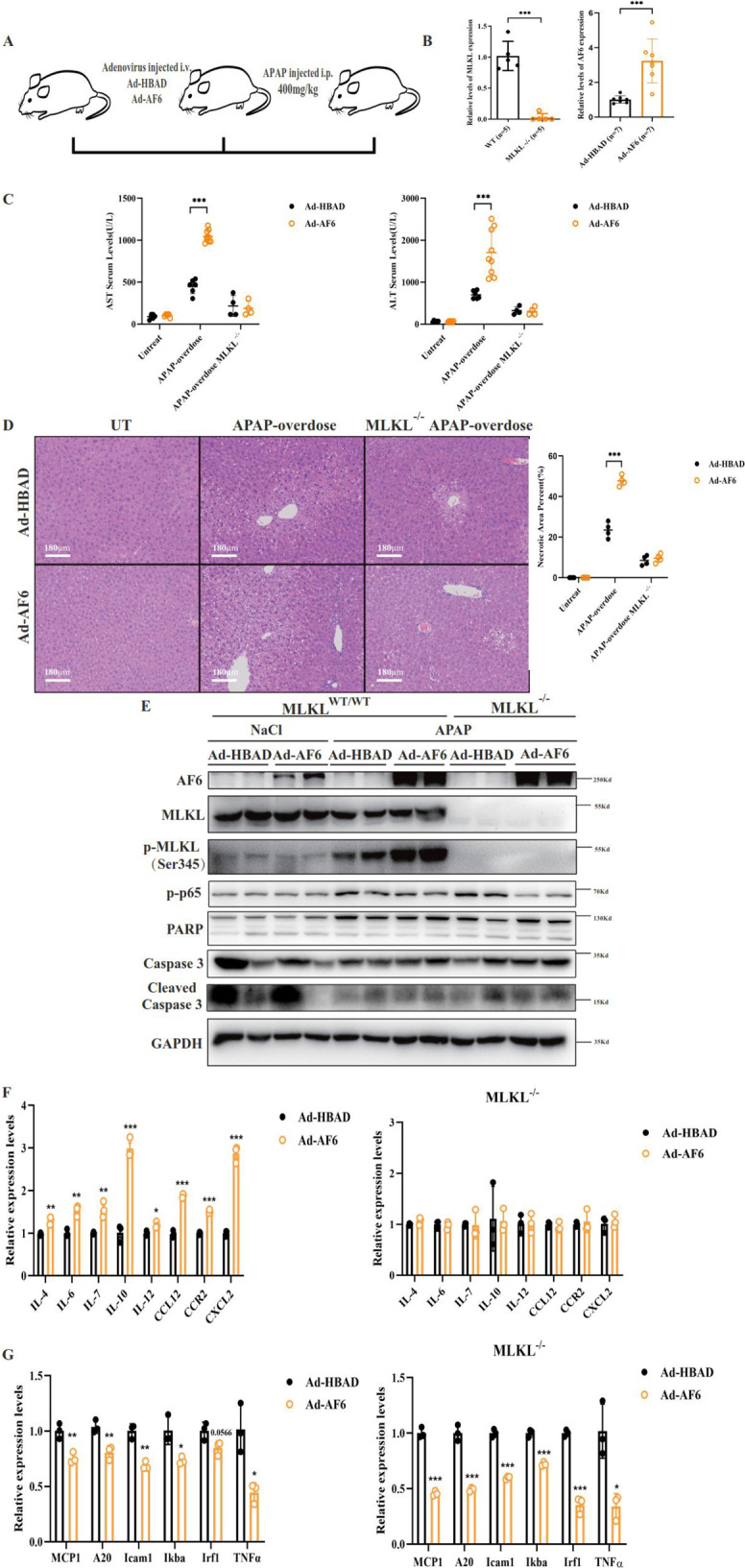
Overexpression of AF6 in MLKL knockout mice did not aggravate the severity of acute liver injury induced by APAP. **A** Work model of Acetaminophen (APAP)-overdose-induced acute liver injury. **B** The effective efficiency of hepatic AF6 overexpression and MLKL knockout at mRNA levels was examined by RT-PCR. **C** The circulating levels of serum AST and ALT were measured after APAP injection in C57/BL mice by kit. **D** HE staining of pathological sections in APAP-overdose induced acute liver injury model and the necrosis area in liver was measured by Photoshop. **E** Immunoblot of proteins relating to cell death and NF-κB signaling pathway in liver after APAP injection. mRNA transcription changes of (**F**) inflammatory cytokines genes and (**G**) NF-κB targeted genes were determined by quantitative RT-PCR. Values were normalized to the mRNA level of the housekeeping gene *36b4* in the respective sample. Data are expressed as mean ± SEM. Pairwise comparisons between groups were conducted using two-tailed non-paired Student’s *t* tests. ns, not significant (*p* ≥ 0.05); *p < 0.05; **p < 0.01; ***p < 0.001.

As a further confirmation of the role of AF6 in in vivo necroptosis, assessed the effect of AF6 overexpression in a mouse model of acute liver injury. Specifically, we systemically infected *Mlkl* KO mice (i.e., lacking any MLKL expression) with Ad-AF6; these mice were then subjected to acetaminophen (APAP) overdose by intraperitoneal injection, inducing a clinically relevant noninfectious animal model of liver injury (Fig. 7a). Notably, APAP-induced hepatotoxicity is the most common cause of acute liver failure in the US [41, 42]. Firstly, we verified AF6 overexpression and *Mlkl* KO in these animals (Fig. 7b). Secondly, we confirmed that AF6 overexpression did not affect the health of the mice or the serum levels of inflammatory factors (Fig. 7c). Finally, following APAP injection, we monitored liver damage and systemic inflammation in the *Mlkl*^*−/−*^ and Ad-AF6 *Mlkl*^*−/−*^ mice. As expected, serum levels of the AST and ALT liver enzymes were elevated (following APAP injection) in APAP-dosed AF6-overexpressing mice, but not in *Mlkl*^*−/−*^ animals and Ad-AF6 *Mlkl*^*−/−*^ mice (Fig. 7c). H&E staining of liver tissues showed markedly pericentral necrosis in Ad-AF6 mice, but not in *Mlkl*^*−/−*^ animals and Ad-AF6 *Mlkl*^*−/−*^ (Fig. 7d). Indeed, as assessed by immunoblotting, the frequency of hepatocyte necroptosis was higher in the former than in the latter, and we did not observe the effect of AF6 on apoptosis in the APAP-induced liver injury model (Fig. 7e). Expression of inflammatory factors and NF-$\kappa$B target genes were also examined in liver. Consistent with previous results, AF6 overexpression significantly suppressed the transcription of NF-KB target genes in APAP-induced polar liver injury in both wild-type and *Mlkl*^*−/−*^ mice (Fig. 7g). However, the enhanced expression of inflammatory factors by AF6 was abolished in *Mlkl*^*−/−*^ mice (Fig. 7f). Our results suggested that AF6 is a novel regulator of MLKL-dependent necroptosis and liver damage in response to noninfectious liver injury. Our studies also suggested that the fatty liver with elevated AF6 levels may be more sensitive to TNF-induced necroptosis and liver cell death. We hypothesize that this increased sensitivity maybe one cause of “fragile” liver and severe inflammation in response to liver damage and disease.

## Discussion

Overall, this work demonstrated that the accumulation of fat in various liver diseases is accompanied by the accumulation of the polar protein AF6. In the present study, we demonstrated that abnormal elevation of AF6 accelerates the progression of necroptosis and is tightly associated with hepatocyte death and lethal liver pathology, including acute liver injury, NASH and SIRS. Mechanistic studies revealed that AF6 directly interacts with RIPK1 and alters the ubiquitination (via K63-linked ubiquitin) of the RIPK1 376 site by regulating the USP21 deubiquitylase enzyme, thereby potentiating necroptosis. Furthermore, *Af6*^*LKO*^ mice were attenuated for liver damage and inflammation (compared to *Af6*^*flox/flox*^ (undeleted) animals) in a variety of in vivo necroptosis-related liver disease models. At the same time, in a SIRS model (induced by dosing with TNFα), adenovirus-mediated hepatic overexpression of AF6 significantly boosted inflammation and increased mortality. In summary, our studies suggested that AF6 is a novel activator of necroptosis in the liver, a regulatory effect that was mediated by changes in the ubiquitination of RIPK1 via the action of USP21. Our studies also suggested that, when maintained on HFHC, animals with elevated levels of AF6 are more sensitive to TNF-induced necroptosis and liver cell death. Future studies must examine why fatty liver results in enhanced cell death, severe inflammation and “fragile” liver. We also showed that hepatic knockdown of AF6 ameliorated necroptosis under severe pathological conditions such as liver injury, NASH and even in SIRS.

Previous studies have shown that AF6 serves as a key adhesion protein for epithelial cells, which in normal tissues must maintain cell-cell adhesions and cellular polarity [5, 6]. AF6 often has been detected as a complex modulator, and loss of AF6 has been shown to be associated with tumor progression and migration. However, recent research, including work by our laboratory, has demonstrated a surprising role for AF6 in other biological processes, including diabetes [9], fat metabolism [43, 44], and mechanotransduction [45]. Our published studies reveal that hepatic accumulation of AF6 is associated with hepatic insulin resistance; this observation contrasts to those in other organs, in which AF6 level typically fall during disease progression. Interestingly, divergent expression of other polarity proteins, including Scribble, also has been reported by other research groups, such that the levels of such proteins are increased in liver but decreased in other organs which was down-regulated in other organs [46, 47]. Thus, the function of polarity proteins in liver appears to be more complex than that in other epithelial tissues. However, our work did not reveal the mechanism whereby AF6 accumulates in liver. Consistent with our previous report, the present study suggested after dosing with OA or PA results in upregulation of the polarity protein AF6 in primary hepatic cells. In combination with other work (data not shown), our study demonstrates that the liver exhibits cell-type-specific regulation of polarity protein expression, in contrast to the case in cells of other epithelial tissues. Further studies are warranted to address this notion. Given that the liver receives blood through two complex blood supply systems, the location and nature in liver of tight junctions and adhesion junction complexes, membrane-bound structure that incorporate AF6, is expected to differ from those in other epithelial tissues. Improved understanding of how the scaffold protein AF6 is associated with the maintenance of liver morphology and hepatic organization is expected to clarify the role of AF6 in these tissue-specific effects.

Differential activation of specific types of programmed cell death may affect outcomes in liver diseases, suggesting in turn, novel opportunities for therapeutic intervention. For a long time, the term apoptosis was thought to be synonymous with programmed cell death. Additionally, given the challenge of detecting the expression of RIPK3 in the liver using existing methods, the occurrence of necroptosis in the liver remains controversial [48]. However, increased expression of RIPK3 has been detected in the livers of both mice and clinical patients [49, 50]. Several previous studies have shown that inhibition of RIPK1 or MLKL can retard the progression of liver disease [19, 51]. In other work, we also have shown that a RIPK1 inhibitor (Nec-1) attenuates the acute liver injury caused by CCl_4_ (data not shown). At the same time, liver-specific knockout of *Ripk3* has been shown to attenuate liver damage and inflammation in some models of liver disease models [22, 50]. MLKL is known to regulate necroptosis during hepatitis through signal transducer and activator of transcription 1 rather than via RIPK3 [52]. Moreover, the microenvironment generated by the cytokines produced by necroptosis in the liver may favor the development of hepatocellular carcinoma instead of cholangiocarcinoma [53]. These results demonstrate that the role of pro-inflammatory necroptosis in the liver remains poorly elucidated. Our RNA-seq data revealed that necroptosis is readily detected in NASH mice maintained on a HFHC diet, a condition under which liver inflammation is apparent. Our study fills in a gap, providing direct evidence that polarity proteins contribute not just to the maintenance of epithelial homeostasis but to the activation of necroptosis. These observations contribute to our understanding of the prominent role that RIPK1 plays in proinflammatory and cell-survival signaling, inducing activation of the NF--κB pathway downstream of the activation of TNF- and TRIF-dependent Toll-like receptors (TLRs) in the liver.

RIPK1 is a master regulator of cell death and inflammation, and undergoes multiple post-transcriptional modifications to determine the protein’s pro-survival or pro-cell-death function. Ubiquitylation and phosphorylation of RIPK1 have emerged as crucial mediators of signal transduction. Notably, ubiquitination restricts RIPK1 death-inducing activity. Previous studies have revealed that the key E3 ligases cIAP and Linear ubiquitin chain assembly complex regulate ubiquitination of RIPK1 at K11 [54], K48 [55], K63 [56], and M1 [57]; these modifications contribute to the activation of the NF-κB and mitogen-activated protein kinase (MAPK) signaling pathways and triggering survival signals. Residue K376 of mRIPK1 has been shown to be critical for Complex I formation [58], while residue K115 is known to be ubiquitinated during TNF-induced necroptosis [59]. Other recent work has shown that ubiquitination of RIPK1 at residue K612 regulating both RIPK’s pro-death kinase activity (following TNFα stimulation) and pro-survival activity in response to TLR signaling [60]. The ubiquitin modifications on RIPK1 serve as anchors to recruit various ubiquitin-binding proteins that hamper the activation of RIPK1 kinase and promote pro-survival signaling. The pro-survival signal mediated by RIPK1 depends on the ubiquitination of the intermediate domain [58], and the absence of ubiquitination at K376 abolishes NF-kB activation, resulting in embryonic lethality [31, 33]. The present work indicated that AF6 binds to the intermediate domain of RIPK1, and that mutation at RIPK1 residue K376 site blocks AF6’s regulation of RIPK1 ubiquitination. Together, these results suggest that AF6 exerts its regulatory role through RIPK1 residue K376, though it remains unclear whether this residue also mediates the transduction of other signals by RIPK1.

The ubiquitination of RIPK1 determines the occurrence of apoptosis and necroptosis. We observed that in the case of prolonged TNFα treatment, the decrease in RIPK1 ubiquitination resulting from AF6 overproduction also promotes the cleavage of PARP and caspase 3, implying the occurrence of apoptosis. Were caspase 8 inhibited, cell death will be transformed the necrosome into a necroptotic complex. AF6 may contribute, in part, to the inhibition of caspase 8; in combination with the simultaneous impairment of K63-linked polyubiquitination of RIPK1, this effect may be sufficient to induce necroptosis in hepatocytes with elevated AF6 levels.

**Fig. 8:**
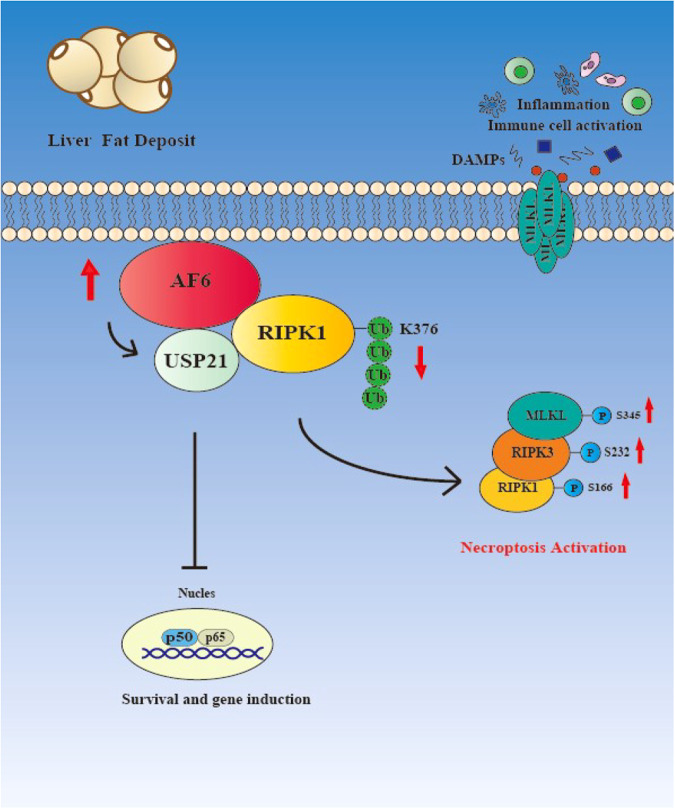
Work model of the role of hepatic AF6 in necroptosis regulation in liver diseases. Our studies indicated that upregulation of the polarity protein AF6 is critical to liver injury and inflammation in liver metabolic diseases. Mechanistically, AF6 appears to regulate the ubiquitination of RIPK1 by directly interacting with RIPK1 and decreasing RIPK1 ubiquitination in a USP21-dependent manner, thereby inducing necroptosis.

In summary, our data suggest that upregulation of the polarity protein AF6 is critical to liver injury and inflammation in the model of NASH and SIRS. Mechanistically, AF6 appears to regulate the ubiquitination of RIPK1 by directly interacting with RIPK1 and decreasing RIPK1 ubiquitination in a USP21-dependent manner, thereby inducing necroptosis (Fig. 8). Our findings identify AF6 as a previously unrecognized contributor to proinflammatory necroptosis. Knockdown of AF6 and proinflammatory necroptosis may serve as potential strategies for new therapeutic interventions against inflammation-associated liver disease.

### Supplementary information

Supplemental Figure 1

Supplemental Figure 2

Supplemental Figure 3

Supplemental Figure 4

Supplemental Figure 5

Supplemental Figure legend

Supplemental Material Original Blots

## Data Availability

The original data used to support the findings of this study are available from the corresponding author upon request.
