# Supplementary figures and images for: Polarity protein AF6 functions as a modulator of necroptosis by regulating ubiquitination of RIPK1 in liver diseases
Source: Cell Death Dis. 2023 Oct 12;14(10):673. doi: 10.1038/s41419-023-06170-8 (PMC10570300; doi:10.1038/s41419-023-06170-8)

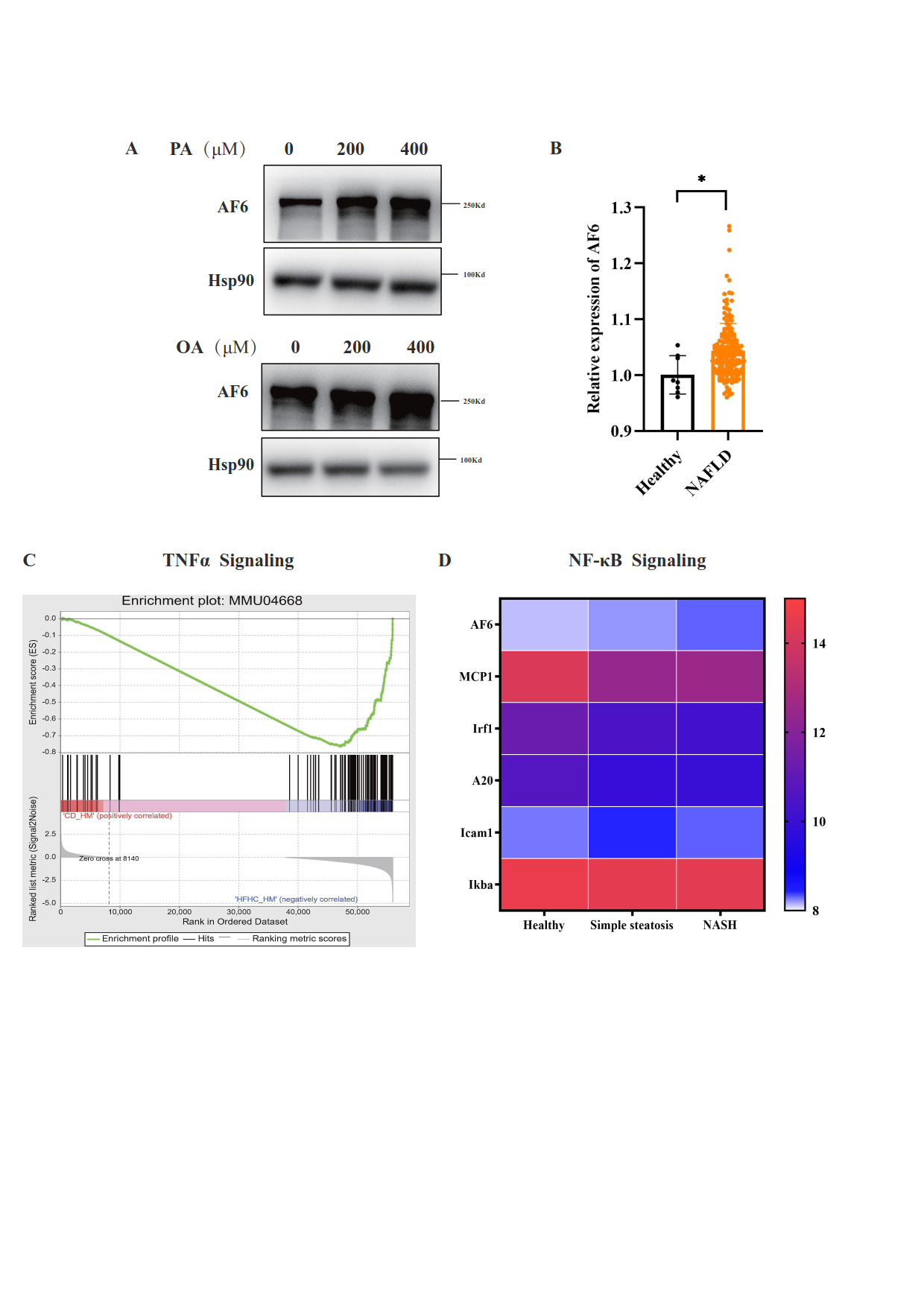

Supplement: Supplementary file 1 — Supplemental Figure 1 [file 41419_2023_6170_MOESM1_ESM.tif]

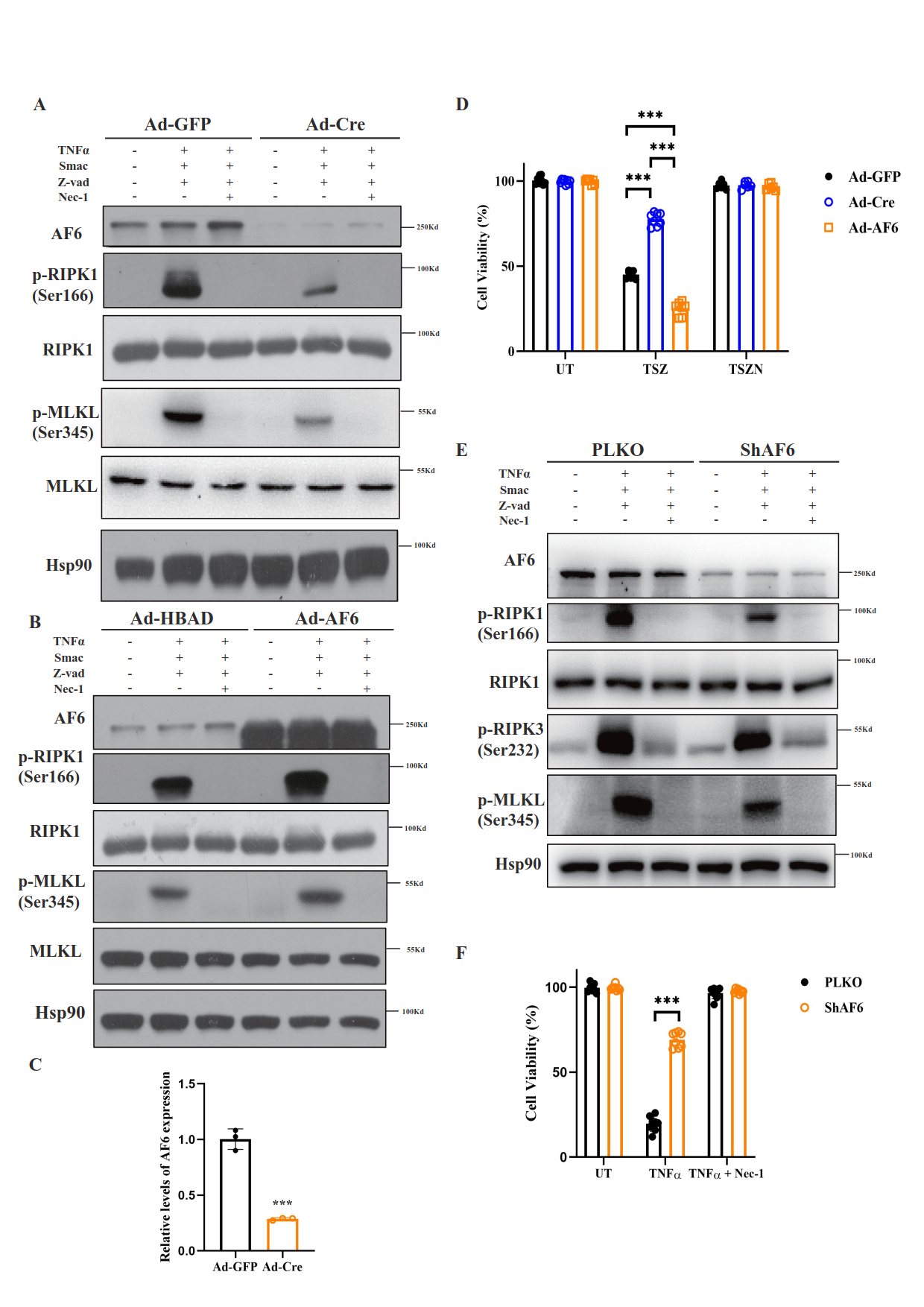

Supplement: Supplementary file 2 — Supplemental Figure 2 [file 41419_2023_6170_MOESM2_ESM.tif]

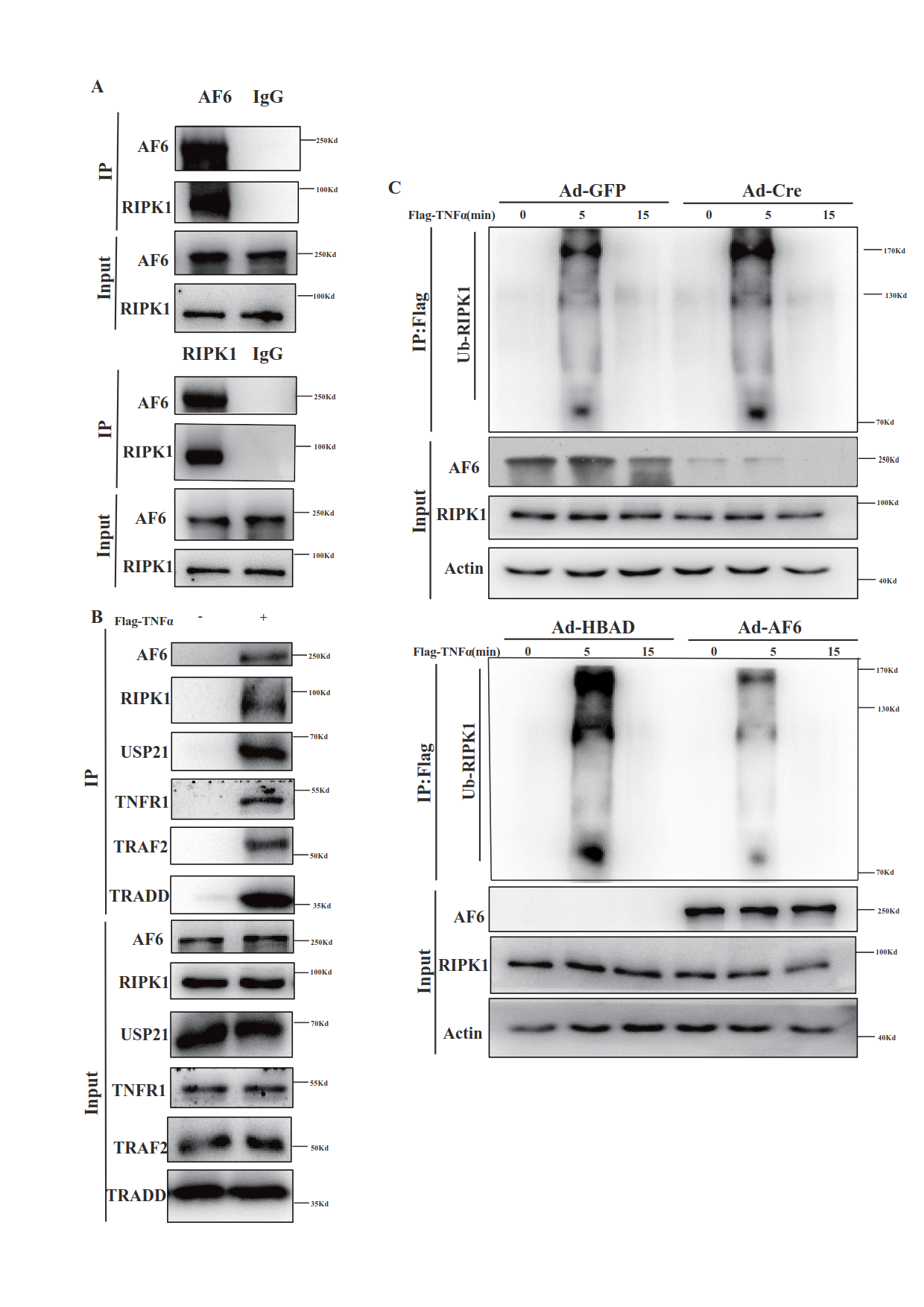

Supplement: Supplementary file 3 — Supplemental Figure 3 [file 41419_2023_6170_MOESM3_ESM.tif]

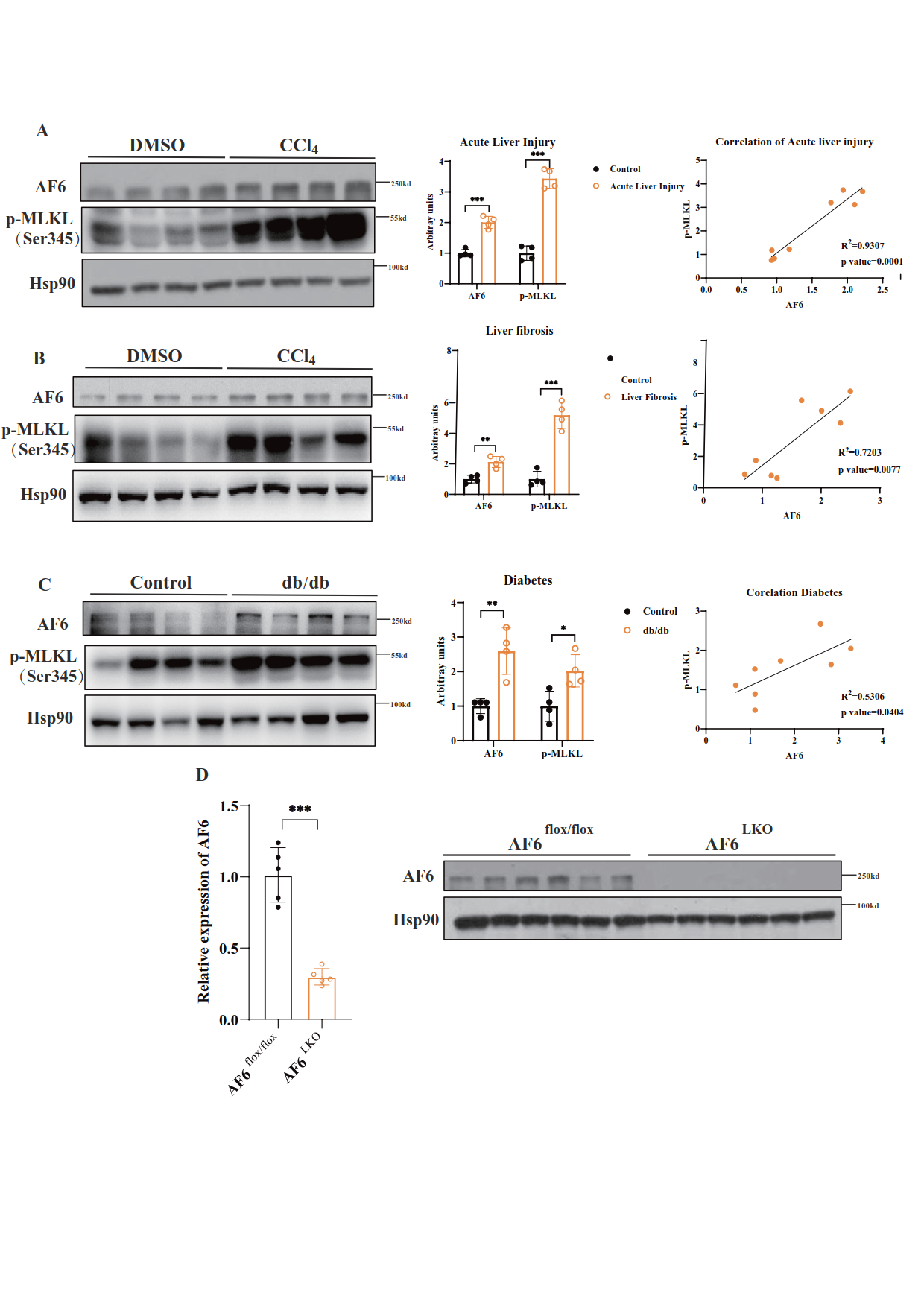

Supplement: Supplementary file 4 — Supplemental Figure 4 [file 41419_2023_6170_MOESM4_ESM.tif]

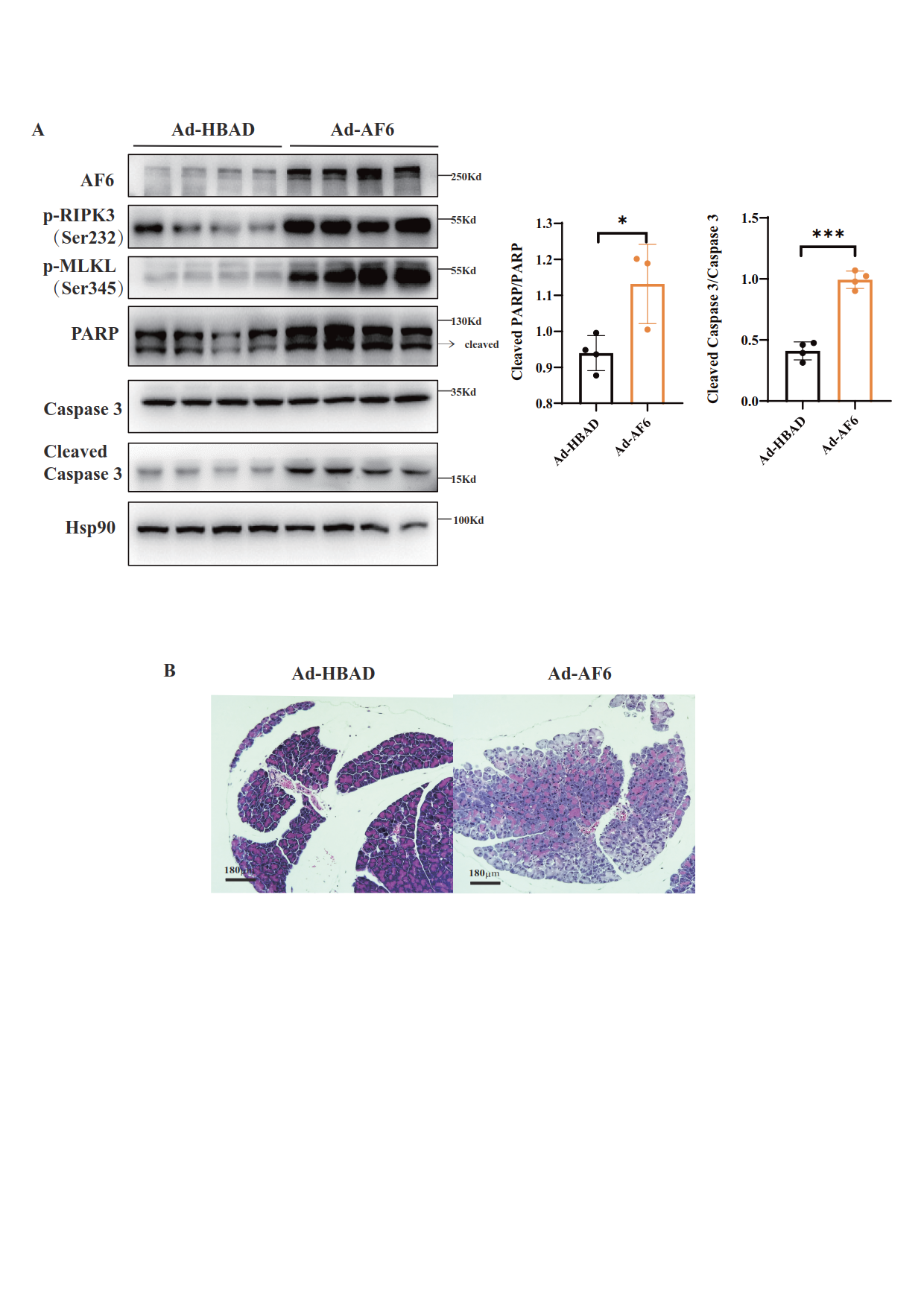

Supplement: Supplementary file 5 — Supplemental Figure 5 [file 41419_2023_6170_MOESM5_ESM.tif]

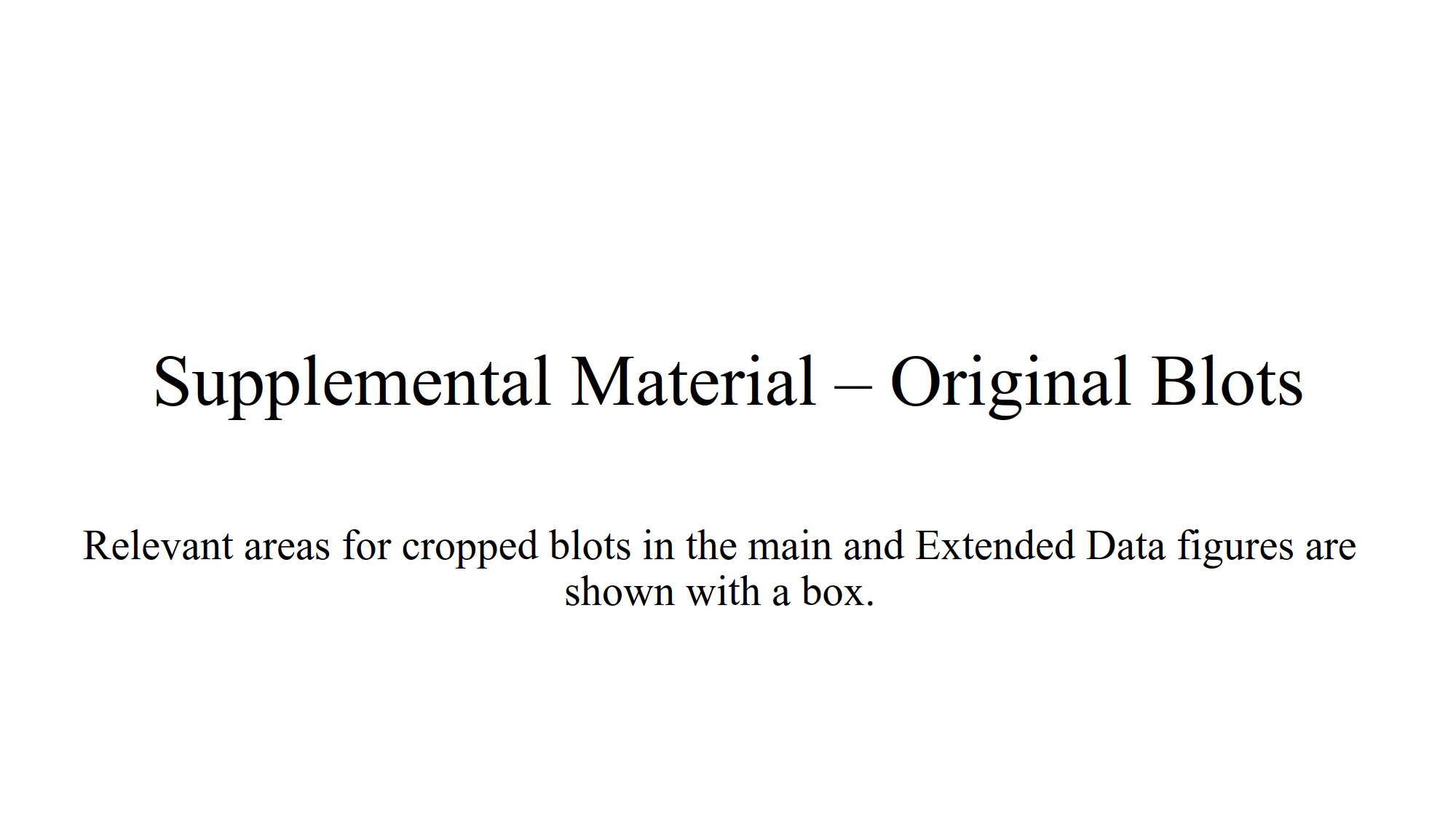

Supplement: Supplementary file 7 — Supplemental Material Original Blots [file 41419_2023_6170_MOESM7_ESM.tif]
