# Supplementary material for: Polarity protein AF6 functions as a modulator of necroptosis by regulating ubiquitination of RIPK1 in liver diseases
Source: Cell Death Dis. 2023 Oct 12;14(10):673. doi: 10.1038/s41419-023-06170-8 (PMC10570300; doi:10.1038/s41419-023-06170-8)
Supplement: Supplementary file 6 — Supplemental Figure legend [file 41419_2023_6170_MOESM6_ESM.docx]

**Supplemental Figure 1. AF6 Expression and necroptosis are up-regulated and correlated in non-alcoholic steatohepatitis. (A)** Immunoblot of AF6 and Hsp90 in *Af6^flox/flox^* primary hepatocytes after palmitic acid (PA) and oleic acid (OA) stimulation for 24 hours.**(B)** Hepatic levels of AF6 in healthy people (n=8) and in non-alcoholic fatty liver diseases(NAFLD) (n=184) patients in GSE135251. **(C)** GESA gene enrichment analysis on TNFα pathway in HFHC induced NASH model. **(D)** GESA gene enrichment analysis on NF-κB signaling pathway in GSE89632. Data are expressed as mean ± SEM. Pairwise comparisons between groups were conducted using two-tailed non-paired Student’s t-tests. ns, not significant (p ≥0.05); *p < 0.05; **p < 0.01; ***p < 0.001.

**Supplemental Figure 2. AF6 exacerbates necroptosis and cell death in vitro.** The levels of key molecules of the necroptosis pathway were determined by immunoblotting primary hepatocytes of *Af6^flox/flox^* mice infected with Ad-GFP and **(A)** or Ad-HBAD and Ad-AF6 adenoviruses **(B)** , L929 PLKO(AF6 normal expression) and ShAF6 (AF6 KD) cells **(E)** and BMDM cells after siRNA transfection **(G)**. **(C)** The effective efficiency of hepatic *Af6* knockout at mRNA levels was examined by RT-PCR. **(D)**Cell viability was assessed in primary hepatocytes of *Af6^flox/flox^* mice infected with Ad-GFP, Ad-Cre, or Ad-AF6 and then grown in the presence of media containing vehicle (untreated; UT), TNFα (50ng/ml), Smac mimetic (100nM) and z-VAD (20μM) (TSZ), or TNFα (50ng/ml), Smac mimetic (100nM),z-VAD (20μM) and Nec-1 (30μM) (TSZN). **(F)**Cell viability was assessed in L929 PLKO or ShAF6 cells grown in the presence of media containing vehicle (untreated; UT), TNFα (50ng/ml), or TNFα (50ng/ml) and Nec-1 (30μM). **(H)** Cell viability was assessed in BMDM cells after SiRNA treatment and grown in the presence of media containing vehicle (untreated; UT), LPS (50ng/ml), CHX (100nM) and z-VAD (20μM) (LCZ), or LPS (50ng/ml), CHX (100nM), z-VAD (20μM) and Nec-1(30μM) (LCZN). Data are expressed as mean ± SEM. Pairwise comparisons between groups were conducted using two-tailed non-paired Student’s t-tests. ns, not significant (p ≥0.05); *p < 0.05; **p < 0.01; ***p < 0.001.

**Supplemental Figure 3. AF6 can interact with RIPK1 and impact its ubiquitination. (A)** Endogenous AF6 bound to endogenous RIPK1 in *Af6^flox/flox^* MEF cells. MEF cells were lysed with NP-40 buffer and immunoprecipitated with control mouse IgG or anti-AF6, anti-RIPK1 monoclonal antibody and A/G-agarose beads for 4 hours at room temperature and immunoblotted as indicated using antibodies against the indicated proteins. **(B)** MEF cells of *Af6^flox/flox^* mice were stimulated with Flag-TNFα (100ng/mL) for 5 min. Then cells were lysed with NP-40 buffer and immunoprecipitated with anti-Flag beads for 2 hours at room temperature and then immunoblotted as indicated using antibodies against the indicated proteins.**(C)** The ubiquitination of RIPK1 in *Af6^flox/flox^* MEF cells infected with Ad-GFP or Ad-Cre or Ad-HBAD or Ad-AF6 were stimulated with Flag-TNFα (100ng/mL) for indicated time. Cells were lysed with NP-40 buffer and immunoprecipitated with anti-FLAG beads for 2 hours at room temperature and then immunoblotted as indicated using antibodies against the indicated proteins. **(D)** MEF cells of  *Af6^flox/flox^* mice were lysed with NP-40 buffer and immunocoprecipitated with mouse anti-IgG, anti-RIPK1 and anti-AF6 monoclonal antibodies and A/G-agarose beads for 4 hours at room temperature and then immunoblotted as indicated using antibodies against the indicated proteins.

**Supplemental Figure 4. Liver knockout of AF6 can slow the progression of various liver diseases through necroptosis.** Immunoblot of proteins AF6 and p-MLKL expression in other liver diseases, such as **(A)** acute liver injury, **(B)** liver fibrosis and **(C)** diabetes. Densitometric analysis was used to quantify band intensities; the protein levels of AF6 and p-MLKL were normalized to that of the housekeeping protein Hsp90 in the respective lanes. Correlation between AF6 and p-MLKL levels was assessed using Graphpad Prism 8.0. **(D)** The effective efficiency of hepatic AF6 knockout at mRNA and protein levels was examined by RT-PCR and immunoblotted as indicated using antibodies against the indicated proteins. Data are expressed as mean ± SEM. Pairwise comparisons between groups were conducted using two-tailed non-paired Student’s t-tests. ns, not significant (p ≥0.05); *p < 0.05; **p < 0.01; ***p < 0.001.

**Supplemental Figure 5. Animal that Over-express of AF6 is sensitive to TNF**$\boldsymbol{\alpha}\mathbf{-}$**induced SIRS in vivo. (A)** Immunoblot of proteins relating to cell death signaling pathway in pancreas after TNFα injection. Densitometric analysis was used to quantify band intensities. The protein levels of cleaved PARP and cleaved caspase 3 were normalized to the housekeeping protein Hsp90 in the respective lanes. **(B)** HE staining of pathological pancreas sections in TNF$\alpha$ induced SIRS model with or without AF6 overexpression. Data are expressed as mean ± SEM. Pairwise comparisons between groups were conducted using two-tailed non-paired Student’s t-tests. ns, not significant (p ≥0.05); *p < 0.05; **p < 0.01; ***p < 0.001.
